# Three assays for in-solution enrichment of ancient human DNA at more than a million SNPs

**DOI:** 10.1101/gr.276728.122

**Published:** 2022

**Authors:** Nadin Rohland, Swapan Mallick, Matthew Mah, Robert Maier, Nick Patterson, David Reich

**Affiliations:** 1 Department of Genetics, Harvard Medical School, Boston, Massachusetts 02115, USA;; 2 Broad Institute of MIT and Harvard, Cambridge, Massachusetts 02142, USA;; 3 Howard Hughes Medical Institute, Boston, Massachusetts 02115, USA;; 4 Department of Human Evolutionary Biology, Harvard University, Cambridge, Massachusetts 02138, USA

## Abstract

The strategy of in-solution enrichment for hundreds of thousands of single-nucleotide polymorphisms (SNPs) has been used to analyze >70% of individuals with genome-scale ancient DNA published to date. This approach makes it economical to study ancient samples with low proportions of human DNA and increases the rate of conversion of sampled remains into interpretable data. So far, nearly all such data have been generated using a set of bait sequences targeting about 1.24 million SNPs (the “1240k reagent”), but synthesis of the reagent has been cost-effective for only a few laboratories. In 2021, two companies, Daicel Arbor Biosciences and Twist Bioscience, made available assays that target the same core set of SNPs along with supplementary content. We test all three assays on a common set of 27 ancient DNA libraries and show that all three are effective at enriching many hundreds of thousands of SNPs. For all assays, one round of enrichment produces data that are as useful as two. In our testing, the “Twist Ancient DNA” assay produces the highest coverages, greatest uniformity on targeted positions, and almost no bias toward enriching one allele more than another relative to shotgun sequencing. We also identify hundreds of thousands of targeted SNPs for which there is minimal allelic bias when comparing 1240k data to either shotgun or Twist data. This facilitates coanalysis of the large data sets that have been generated using 1240k and Twist capture, as well as shotgun sequencing approaches.

The strategy of using artificially synthesized oligonucleotides that are free in solution as baits to fish out complementary sequences in a DNA library (Gnirke et al. 2009) has been transformative for studying ancient DNA. Under appropriate chemical and temperature conditions, these baits hybridize to targeted molecules so other molecules can be washed away, allowing the bound molecules to be isolated, released, and sequenced. Enrichment has allowed researchers to achieve orders of magnitude of enrichment for sequences addressing important scientific questions.

The most common application of enrichment in the genetics community has been to target the ∼2% of the genome in coding sequences of genes (the “exome”) (Gnirke et al. 2009; Teer and Mullikin 2010). When whole-genome sequencing at high coverage was still prohibitively expensive, exome sequencing dropped the cost for surveillance of the coding regions for mutations causing rare diseases to affordable levels. In ancient DNA analysis, the benefits of enrichment are even greater (Carpenter et al. 2013). Not only is a tiny fraction of the genome in practice relevant for the great majority of analyses, but typically only a small proportion of molecules in an analyzed library come from the individual of interest because of microbial contamination. For example, of more than 3000 ancient individuals for which our research group published genome-wide data by the end of calendar year 2021, about half had <10% human DNA. Whole-genome sequencing in such cases is prohibitively expensive for all but the most important samples.

As an illustration of the power of in-solution enrichment, consider studying an ancient individual at a set of about 600,000 single-nucleotide polymorphism (SNP) positions that have been genotyped in diverse modern human populations. Only one in about 100 ancient DNA sequences mapping to the human genome will overlap these positions. If a DNA library is only 1% human, the proportion of sequences that will be informative for analysis will only be about one in 10,000. Thus, if about 400 million DNA sequences are read from a library, which is a typical number used to produce a ∼30× whole-human genome from modern DNA, at most about 40,000 informative SNPs will be retrieved. In contrast, 25 million sequences from the same ancient DNA library after in-solution enrichment can provide coverage on nearly all targeted SNP positions by multiple unique molecules, allowing accurate inferences about population history at much lower cost.

In-solution enrichment for ancient human DNA libraries was pioneered between 2010 and 2013 in studies that enriched for mitochondrial DNA (Maricic et al. 2010; Fu et al. 2013), nearly all of the unique sequences of Chromosome 21 (Fu et al. 2013), and all or part of the exome (Burbano et al. 2010; Castellano et al. 2014). The great majority of ancient DNA SNP enrichment data sets published to date have used the “1240k reagent,” for which data were first published in 2015 and which targets slightly fewer than 1.24 million SNPs chosen to be particularly valuable for studying variation among modern human populations (Fu et al. 2015; Haak et al. 2015; Mathieson et al. 2015). It has proven highly effective and, as of October 2022, has been used to generate genome-wide data on more than 7000 individuals published in more than 90 papers and constituting >70% of the genome-wide ancient human DNA data sets in the literature (compiled at https://reich.hms.harvard.edu/allen-ancient-dna-resource-aadr-downloadable-genotypes-present-day-and-ancient-dna-data). The large body of data produced using the 1240k reagent has also created a legacy data set: Any future enrichment data benefits by targeting the same set of sites, which can then be coanalyzed with existing data. However, the 1240k reagent has limitations, including variability in effectiveness of enrichment of targeted SNPs and bias toward capturing some alleles more than others at the same sites, leading to technical artifacts when such data are coanalyzed with other data types such as random “shotgun” sequencing data. Population genetic analyses often restrict analyses to 1240k data only or to shotgun data only, excluding key datapoints generated using the other strategy.

A challenge with the 1240k assay is that many ancient DNA laboratories have not been able to practically access the technology. Although the bait sequences were fully published in 2015, secondary distribution of the physical reagent was not permitted by the company that synthesized the oligonucleotides, and resynthesis was prohibitively expensive on a per-reaction basis for laboratories interested in using the assay on a scale of fewer than hundreds of samples. To make it possible for any ancient DNA researcher to carry out in-solution SNP enrichment, in 2021 two companies, Daicel Arbor Biosciences and Twist Bioscience, made available in-solution enrichment assays targeting the core panel of 1.24 million SNPs and additional content. Here, we describe a systematic comparison of all three assays on a common set of 27 ancient DNA libraries with low to high human DNA content (Table 1). In the interests of providing an independent assessment, our paper has not been reviewed by the companies that generated the assays.

**Table 1.**
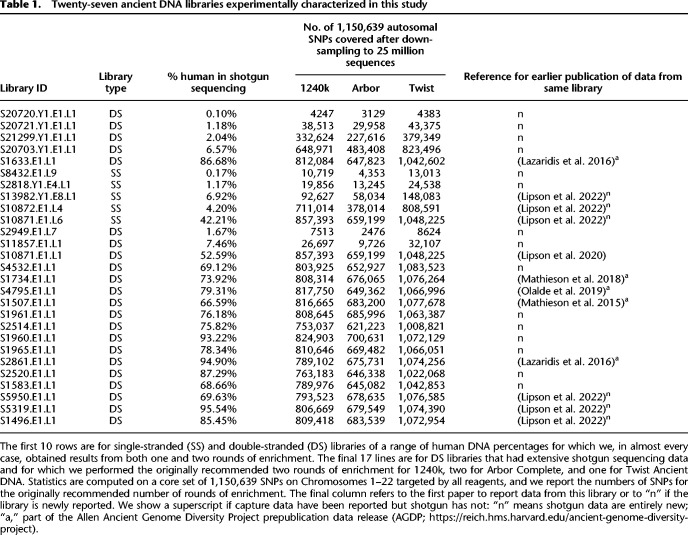
Twenty-seven ancient DNA libraries experimentally characterized in this study

## Results

### Design of the three reagents

In the original “1240k” design (Fu et al. 2015), each SNP was targeted by four probes of 52 bp. To reduce bias toward one allele or the other, two probes abutted but did not overlap the SNP from either direction. Another two probes were centered on the SNP, each with an alternative allele (again with the aim of reducing bias). The probes were appended on one side by an 8-bp universal flanking sequence and the 60-bp oligonucleotides printed on Agilent 1 M custom arrays. The baits were then cleaved, amplified, and biotinylated in preparation for enrichment (Fu et al. 2013).

The 1,233,013 SNPs in the reagent (the count that remained after filtering) were chosen to achieve a variety of purposes, which are summarized in Table 2 and in the original publications (Fu et al. 2015; Haak et al. 2015; Mathieson et al. 2015). The reagent aimed to enrich for all the SNPs in the Affymetrix Human Origins genotyping array (Patterson et al. 2012) that has now been used to publish data on about 8900 present-day people from approximately 810 human populations worldwide. It enriched for SNPs on the Illumina 650Y genotyping array, part of a family of similar Illumina arrays whose content was optimized for genome-wide association studies and that has been widely used in genome-wide studies of human history. It enriched for SNPs on the Affymetrix GeneChip Human Mapping 50K Xba Array, tens of thousands of SNPs on the X Chromosome to enable comparative studies of male and female history, and tens of thousands of SNPs on the Y Chromosome to allow high-resolution determination of haplotypes. Finally, it enriched for SNPs of phenotypic interest as identified through association studies or scans for signals of natural selection, or through being found within particularly important loci. In practice, 1240k enrichments have often been performed with spiked-in probes that also enrich for mitochondrial DNA (Maricic et al. 2010; Fu et al. 2013).

**Table 2.**
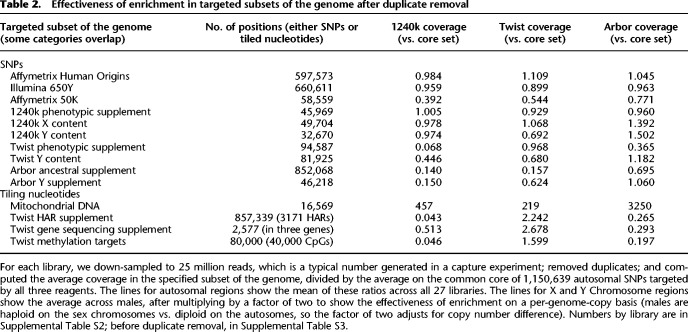
Effectiveness of enrichment in targeted subsets of the genome after duplicate removal

For the Daicel Arbor “myBaits Expert Human Affinities” reagent, the oligonucleotide bait design is proprietary, and we do not have access to the technical details. Several modules are available (https://arborbiosci.com/genomics/targeted-sequencing/mybaits/mybaits-expert/mybaits-expert-human-affinities/). The “Prime Plus” reagent targets the same SNPs as the 1240k reagent and a Supplemental set of 46,218 Y Chromosome SNPs. The “Complete” product adds 852,068 transversion polymorphisms (“Ancestral Plus”) discovered as variable in archaic humans and validated as polymorphic in present-day humans (https://arborbiosci.com/wp-content/uploads/2021/03/Skoglund_Ancestral_850K_Panel_ Design.pdf). These sites were chosen to reduce bias in population genetic analysis as the primarily Eurasian ancestry of the individuals in whom SNPs are discovered can skew statistics when studying African population history (Bergström et al. 2020). Transversion SNPs are also valuable for analyzing ancient DNA libraries not enzymatically treated to remove ancient DNA damage. All the Arbor reagents also include baits to enrich mitochondrial DNA. We characterized the “Arbor Complete” reagent, which after accounting for the intersections of various SNP panels constitutes 2,131,299 SNPs.

For the Twist Bioscience “Twist Ancient DNA” reagent, a single 80-bp probe was centered on each targeted SNP. To avoid bias toward one allele or another, the nucleotide at the position of the SNP was chosen randomly as one of the two alleles not represented in the actual SNP. The reagent was built around a core of 1,200,343 1240k SNPs (all 1240k SNPs on Chromosomes 1–22 and X). It replaced the 32,670 1240k Chromosome Y SNPs with 81,925 chosen to provide improved haplogroup resolution. It also added 94,586 phenotypically relevant targets chosen to target SNPs that were significantly associated to phenotypes in genome-wide association studies in large sample sizes (Watanabe et al. 2019), or as likely to have been affected by natural selection (Speidel et al. 2019), or as possibly implicated in rare disease (Landrum et al. 2020), or as useful for computing heritability of complex traits (Supplemental Text S1; Weissbrod et al. 2020). These SNPs were only added if they were not in strong linkage disequilibrium with the core 1240k set (Supplemental Text S1; Supplemental Data 1). The Twist reagent also targeted non-SNP locations: 857,339 bp in 3171 human accelerated regions (HARs); 2577 bp in three genes relevant to α-thalassemia, β-thalassemia, and favism; and 40,000 CpG dinucleotides for which methylation rates correlate to human age (Supplemental Text S2). After filtering to probes that designed well, the final reagent included 1,434,155 probes targeting 1,352,535 SNPs, of which 1,352,529 (all but six) were included in our bioinformatics analysis. A mitochondrial panel from Twist can be added to the bait pool.

### Empirical characterization of the three assays

We experimentally characterized assay performance in 27 libraries (Table 1) on which we performed 109 enrichment experiments. We report data on 12.2 billion merged sequences obtained for the enrichment experiments and 43.3 billion merged sequences from shotgun sequencing (Supplemental Table S1).

1. For 10 libraries (five double-stranded and five single-stranded) of a range of complexities and percentages of endogenous human DNA (from 0.1%–87%), we performed 58 = 10 × 6 − 2 enrichment experiments (the two most complex libraries were not captured for two rounds for Twist Ancient DNA). We deeply sequenced capture products both after the first and second rounds of sequencing, with a median of 95 million merged reads per experiment.
2. For 17 double-stranded libraries (15 of which had high percentages of human DNA), we performed deep shogun sequencing (in 14 cases to more than 20× coverage) (Table 1; Flegontov et al. 2019; Gokhman et al. 2020; Lipson et al. 2020, 2022). We performed 51 = 17 × 3 enrichments on these libraries with the settings specified in the recommended protocols for each assay at the time we began this study: two rounds of capture for 1240k and Arbor Complete and one round of capture for Twist Ancient DNA. We sequenced the enriched products to a median of 104 million merged sequences.

### Variation in effectiveness of enrichment in different parts of the genome

Table 2 shows the mean coverage in different subsets of the genome relative to the average at the core set of 1,150,639 autosomal SNPs. To assess coverage, we use number of sequences obtained before removal of duplicated sequences as our goal is to study the effectiveness of enrichment. Supplemental Table S2 shows results on a per-library basis, whereas Supplemental Table S3 shows an alternative version of Table 2 before duplicate removal (qualitative findings are very similar). Supplemental Data 1 provides results for each of the 1,352,529 Twist Ancient DNA SNP targets (along with information on why each SNP was targeted). Supplemental Data 2 provides detailed results on each nucleotide of the 40,000 CpGs targeted by the Twist assay. Supplemental Data 3 covers each nucleotide in the 3171 HARs targeted in the Twist assay. Supplemental Data 4 covers each nucleotide that the Twist assay targeted for sequencing (in three genes). Supplemental Data 5 includes lines for 10.4 million alignable nucleotides on the Y Chromosome. Supplemental Data 6 reports results for the 16,569 nucleotides of mitochondrial DNA.

**Figure 1.**
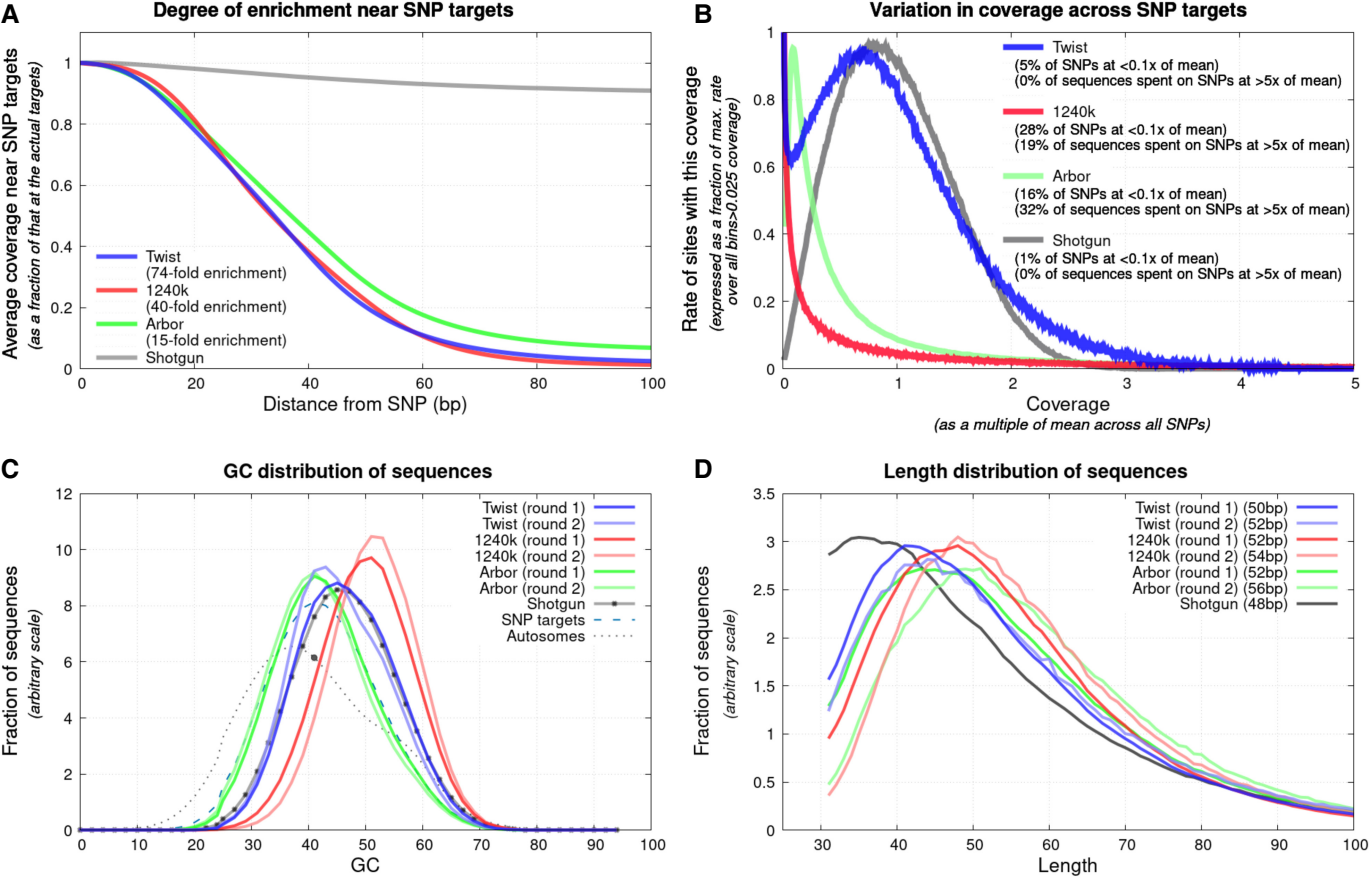
Characterization of enrichment. (*A*) Degree of enrichment as a function of distance from 1,150,639 targeted autosomal SNPs (position 0) for the 15 high-coverage libraries at the bottom of Table 1; enrichment at the SNP relative to positions 100 bp away is shown in the legend. (*B*) Variation in coverage across SNP targets for the same libraries. (*C*) Proportion of nucleotides that are guanine or cytosine (GC) has a downward bias relative to the unenriched library for Arbor, upward for 1240k, and little bias for Twist Ancient DNA; this analysis uses data from the first 10 libraries in Table 1 with full results from both rounds of capture. (*D*) All assays preferentially enrich longer molecules, with the least length effect for Twist Ancient DNA (medians in legend, 10 libraries of data). All plots reflect data before removal of duplicated sequences as our goal is to study effectiveness of enrichment on a per-molecule basis.

All three assays enrich not only for the targeted content but also for other positions, usually within dozens of nucleotides on either side of explicitly targeted content (Fig. 1A). To obtain a better understanding of the patterns of enrichment near targeted locations, we annotated all 81.2 million SNPs in the 1000 Genomes Project data set (The 1000 Genomes Project Consortium 2015) by the coverage relative to the 1240k autosomal SNP targets. Researchers wishing to choose such nontargeted SNPs for inclusion in their analyses can select them based on the information in this set of files (Supplemental Data 7, downloadable by chromosome). All reagents effectively enrich not just the target SNPs, but hundreds of thousands of polymorphic positions nearby. For example, we identified approximately 130,000–170,000 SNPs that were enriched to ≥50% of the autosome-wide average coverage and that had a minor allele frequency ≥5% in at least one 1000 Genomes Project continental population (Table 3).

**Table 3.**
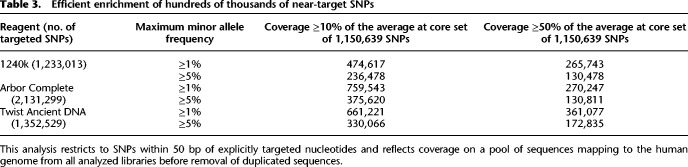
Efficient enrichment of hundreds of thousands of near-target SNPs

### The Twist Ancient DNA assay shows the greatest homogeneity in enrichment

For both shotgun sequencing and the Twist Ancient DNA assay, histograms of SNP coverage are centrally peaked, indicating homogeneous representation of targeted positions (1% of SNPs have coverage <0.1× of the mean for shotgun sequencing; 5%, for Twist) (Fig. 1B). In contrast, we observe uneven enrichment for Arbor (16% of SNPs with coverage <0.1 of the mean) and 1240k (28%). The poor enrichment of 1240k for several hundred thousand SNPs explains why to date, even high-complexity libraries sequenced to multiple-fold average coverage almost never had more than 900,000 targeted SNPs covered at least once, despite there being 1.15 million autosomal targets.

Further evidence for more homogeneous enrichment for Twist Ancient DNA than for the other two assays comes from the proportion of guanines and cytosines in sequenced molecules, which is similar for Twist data and shotgun data, whereas Arbor Complete data shows a downward bias and 1240k an upward bias (Fig. 1C; Supplemental Figs. S3, S4). The shift of the GC-distribution curves is always stronger away from the shotgun data for the second round of enrichment, showing additional biases in the second round. Although Arbor and, to a lesser extent, Twist show a downward shift, 1240k shows a strong upward shift, which we hypothesize reflects a combination of shorter probes and higher hybridization and stringent wash temperatures. The Twist Ancient DNA data also show less of a bias toward an increase in the length of molecules compared with the other methods (Fig. 1D; Supplemental Figs. S2, S4).

**Figure 2.**
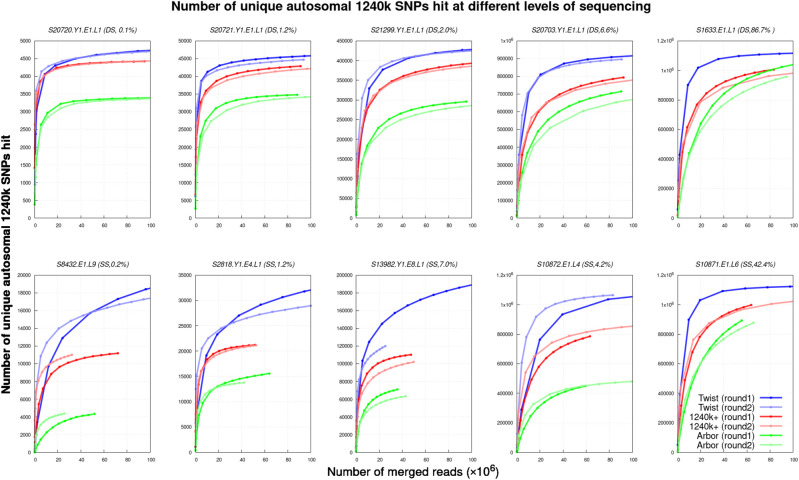
Performance of the three assays over a range of sequencing depths. For 10 libraries (five double-stranded [DS], and five single-stranded [SS] libraries) with varying percentages of human sequences before enrichment (0.1%–86.7%), we show the number of unique SNPs at different levels of sequencing depth (based on down-sampling). For a typical amount of sequencing of a capture experiment (25 million merged sequences), and after removal of duplicated sequences, the Twist Ancient DNA assay always enriches for more SNPs than the other two assays. For most experiments, more SNPs are retrieved after one round of enrichment than after two. We did not perform the two-enrichment-round Twist Ancient DNA experiment for the two libraries with the highest endogenous content (S1633.E1.L1 and S10871.E1.L6).

As expected from its greater homogeneity in enrichment, Twist Ancient DNA achieves consistently higher genome-wide coverage when measured by the number of SNPs covered at least once, when we downsample our data to an amount of sequencing (25 million read pairs) that is typical for such experiments (Table 1; Fig. 2; Supplemental Fig. S1). Compared with 1240k data, the average increase in targeted SNP count is 1.21×, and compared with Arbor Complete, it is 1.46×.

The increased yield for Twist Ancient DNA relative to the other assays is particularly apparent for low-complexity and single-stranded libraries, the condition for which we optimized the Twist Ancient DNA experimental conditions. However, the Twist Ancient DNA assay also outperforms the 1240k assay for double-stranded libraries, which is the condition for which we optimized the 1240k enrichment several years ago with the goal of maximizing SNP coverage and minimizing sequencing costs. For the Arbor Complete experimental settings, we performed no optimization; instead, we used the protocol recommended to us by the manufacturer before product launch, which differs from the one in the online manual. Better enrichment performance (perhaps much better) could likely be achieved with the Arbor Complete reagent if multiple rounds of optimization in experiments were performed such as we performed for Twist Ancient DNA and 1240k. The correct lessons to take from these results are that the Arbor Complete reagent is effective and that these results place a minimum bound, not a maximum, on its utility.

A feature of all three enrichment strategies is the similar genome-wide coverage obtained from one and two rounds of sequencing when a typical amount of data is collected (around 25 million sequences). This is the case even though the proportion of sequences overlapping targets is much higher after two rounds of enrichment (average of 10× higher for the experiments in Fig. 2; Supplemental Table S1). The explanation is that the number of molecules typically sequenced after enrichment is far larger than the number of targeted positions. Thus, even with the relatively small proportions of molecules hitting targets after one round of enrichment, we in practice obtain sequences that cover the great majority of the targeted positions. Because each round of enrichment requires resource expenditure, we recommend that standard practice for all three assays should be to carry out just one round of enrichment.

Our approach of comparing results at 1,150,639 autosomal SNPs common to all three assays in theory underestimates the effectiveness of assays that target more sites (especially Arbor Complete and, to a lesser extent, Twist Ancient DNA). In practice, however, this is not a serious concern in comparing assay effectiveness, as for our recommended setting of a single round of enrichment, the great majority of sequenced molecules miss targets (Supplemental Table S1), and the rate of molecules hitting targets, but not the ones we are using for comparison, is small relative to this off-target number. Correcting for this by removing these “off-target-but-not-really-off-target” sequences from the count would hardly bias assessments of efficiency.

### Addressing technical bias that can arise owing to coanalysis of data from different sources

Biases associated with alignment and enrichment can affect population genetic analysis, causing data from two ancient DNA libraries processed using the same enrichment protocol to appear to have genetic affinities to each other even though the truth is that the individuals from whom the libraries were obtained do not have distinctive relatedness. Concerns of this type have meant that, in practice, for population genetic analyses, researchers have often restricted their analyses to in-solution enrichment data using the 1240k assay or shotgun data, creating a challenging situation in which two disjoint data sets have been built up in the community that are difficult to coanalyze. Even if a technology is more accessible to the community and even if it is more efficient at capturing all targeted positions than the established 1240k enrichment assay, its practical value could be limited if it is difficult to coanalyze with data from other methods.

**Figure 3.**
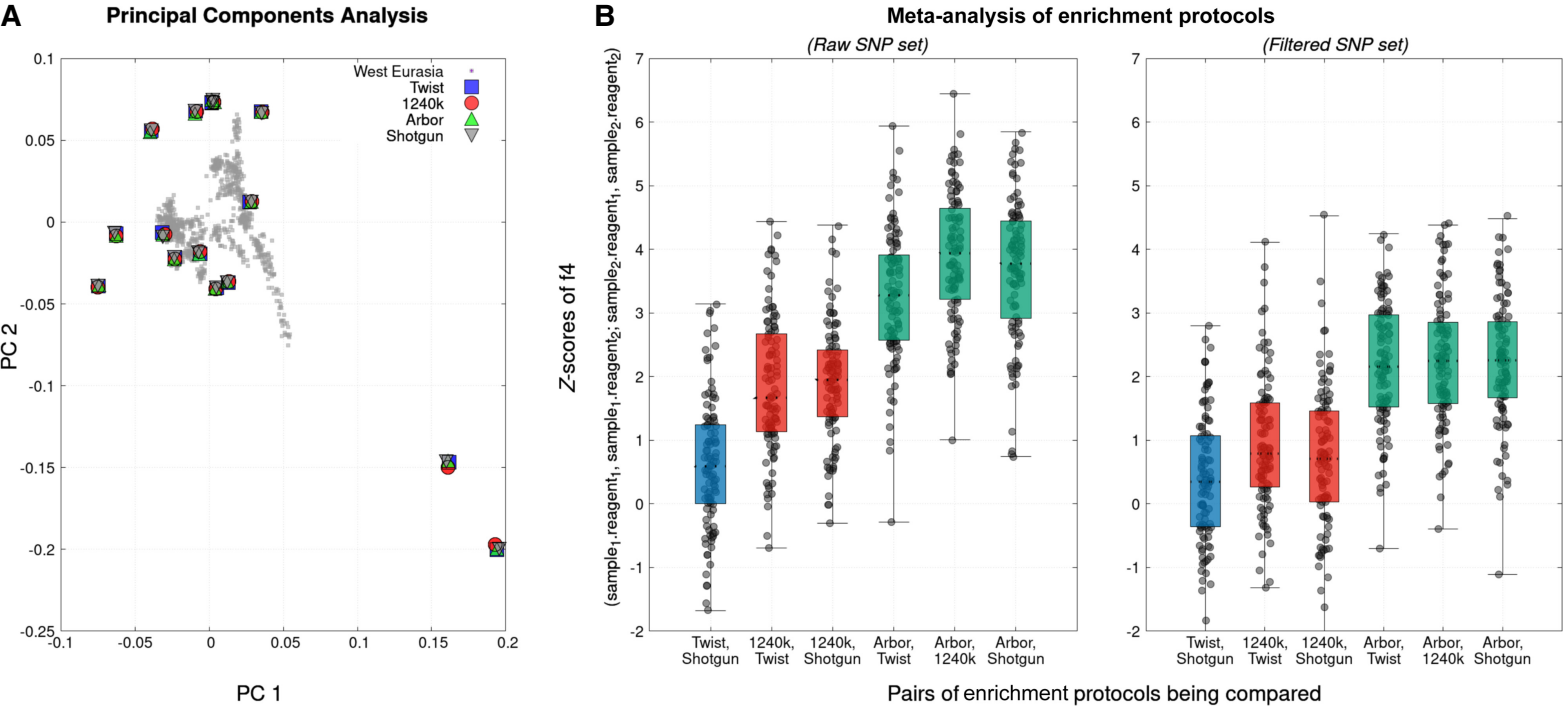
Population genetic effects of enrichment and an effective filter for reducing bias. (*A*) Projection of data from 15 libraries in the last rows of Table 1 onto a PCA of modern West Eurasians (gray squares) shows nearly identical positions regardless of data source. (*B*) We compute symmetry statistics of the form *f*_4_(library 1 − reagent 1, library 1 − reagent 2; library 2 − reagent 1, library 2 − reagent 2) and plot *Z*-scores for all 105 = 15 × 14/2 pairwise comparisons of the libraries (box-and-whisker plots show range, 25th and 75th percentiles, and mean). The statistics involving Arbor Complete are shown in green; remaining comparisons involving 1240k are shown in red; and the Twist–shotgun comparison is in blue. We show results both for all SNPs targets (*left*) and after applying the bias filter retaining a subset of 42% of autosomal SNPs (*right*). Results for this figure reflect data after removal of duplicated sequences.

To explore how bias might affect our results, we first projected data from the 15 libraries at the bottom of Table 1 onto a principal component analysis (PCA) of genetic data from diverse present-day West Eurasian people (Fig. 3A). All data from the same individuals plot at the same position, as in the first publication of Twist Ancient DNA data, which also showed that the two data types were compatible for detecting family relatedness (Fowler et al. 2022).

To explicitly study population genetic biases associated with coanalysis of data generated on different platforms, for each of the 15 high-coverage libraries, we identified all SNP positions that were likely to be heterozygous based on observing both at least one sequence matching the reference allele and at least one matching the variant allele. For each SNP, we counted all additional reference and variant sequences beyond those used in identifying the heterozygous positions; if there are no biases, we expect 50% of these sequences to match the reference variant. We implemented an expectation maximization (EM) algorithm that uses these counts to estimate the distribution of reference bias for all SNPs after correcting for limited sample size (if variation in the reference bias owing to sampling effects is not corrected for, we will infer more apparent variation in reference bias than is, in fact, the case) (Supplemental Text S2).

**Figure 4.**
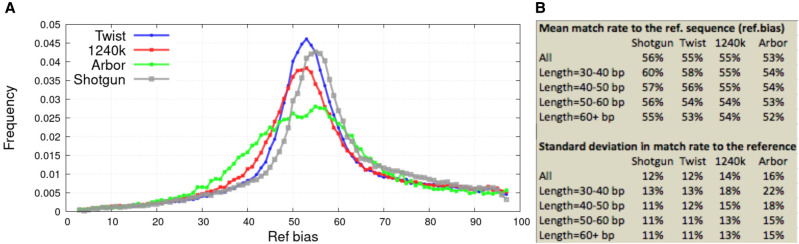
Variation in reference bias across SNPs. (*A*) All analyses are based on sequences from loci ascertained as highly likely to be heterozygous, corrected for stochastic error in the estimates using the expectation maximization (EM) algorithm described in Supplemental Text S2. (*B*) Mean and standard deviation of EM-corrected distributions stratified by sequence length (longer sequences align more reliably so have less bias). Results for this figure reflect data before removal of duplicated sequences.

We observe a rate of matching to the reference allele that is greater than the 50% expected in the absence of reference bias, for all methods of data generation (Fig. 4A). This reflects the fact that when sequences perfectly match the reference genome sequence, they will have a higher probability of aligning with high mapping quality and thus of passing the mapping filters used to allow sequences into analysis (Günther and Nettelblad 2019; Martiniano et al. 2020). However, reference bias affects shotgun data just as much as enrichment data, even after controlling for sequence length (Fig. 4B), and is not the focus of this study. The unique issue for enrichment data is the wider variation in reference bias across SNPs, reflecting the fact that any enrichment technology may be somewhat better at enriching for one allele or another at a particular SNP (Fig. 4B). Such skews specific to a technology are expected to cause data generated from two libraries processed by the same technology to have artifactual affinity. However, the magnitude of this effect varies across the three enrichment methods. The largest variation in reference bias is for the Arbor data, which has a standard deviation of 18% around the mean for 40- to 50-bp sequences, compared with 15% for 1240k and 12% for Twist, which is hardly larger than the 11% seen for shotgun data (Fig. 4A,B).

To study these artifactual attractions, we computed statistics of the form *f*_4_(library 1 − assay 1, library 1 − assay 2; library 2 − assay 1, library 2 − assay 2). If there are no technical biases, such statistics are expected to be zero, as data from each library should be symmetrically related to that from all other libraries. If there are technical biases, we expect positive values reflecting greater-than-random co-occurrences of alleles from two libraries processed using the same technology. Figure 3B (left) computes a *Z*-score for the deviation of these *f*_4_-statistics from zero based on a block jackknife standard error; for the one-sided test appropriate here, *Z* > 1.7 corresponds to *P* < 0.05, and *Z* > 3.1 corresponds to *P* < 0.0001 (Patterson et al. 2012). The statistics are most positive (mean Z of three to four) for comparisons involving Arbor Complete captured SNPs, suggesting the strongest technical bias for this data type, and are consistent with the evidence that Arbor data have the largest standard deviation in reference bias across SNPs, as shown in Figure 4B. The statistics are also large (mean *Z* almost two) for statistics comparing 1240k to Twist Ancient DNA or shotgun data, as expected from the empirical observation of difficulty of coanalyzing these two data types. Bias is minimal for Twist Ancient DNA comparisons to shotgun data (mean *Z*-score of around 0.6 with almost all *Z*-scores between negative two and two).

Although the reduced allelic bias associated with the data produced by the Twist Ancient DNA assay highlights its coanalyzability with shotgun data, it does not solve the challenge of coanalyzability with 1240k data. We therefore set out to identify a subset of SNPs with less susceptibility to such bias. To do this, we mined data from 488 libraries for which we had shotgun data at a median of 5× coverage and also good 1240k data (much of this data set is available as a prepublication data release at https://reich.hms.harvard.edu/ancient-genome-diversity-project). We used imputation with GLIMPSE (Rubinacci et al. 2021) to infer diploid genotypes at each SNP location using a previously described protocol (Patterson et al. 2022) and counted rates of sequences matching to the reference and variant allele in all individuals for which the posterior probability of being heterozygous was >0.9 at a given SNP. We restricted to 42% of autosomal SNPs where the difference in rates of matching to the reference allele for shotgun data and 1240k data was empirically <4% in the pooled reads over 488 libraries (this set of SNPs is specified as a column in Supplemental Data 1). Figure 3B (right) shows that the mean *Z*-scores for all *f*_4_-symmetry statistics comparing libraries that are shotgun sequenced, libraries enriched using 1240k, and libraries enriched using the Twist Ancient DNA assay are between zero and one after restricting to this set of SNPs. We have not attempted to optimize the filter further, and the fact that even such a simple filter has such a meaningful effect suggests there is substantial room to make it better (reducing bias to a greater extent while filtering fewer SNPs). The demonstration of the filter also suggests a solution to the problem that has been a long-standing challenge for ancient human DNA studies: the difficulty of coanalyzing shotgun and 1240k enrichment data. Applying a filter like this has the potential to make data from diverse sources—1240k and shotgun and Twist—coanalyzable even for sensitive population genetic analyses.

## Discussion

We have compared three in-solution assays for enriching ancient DNA libraries and found all three to be highly effective for enriching for many hundreds of thousands of SNP targets.

The 1240k assay has the advantage of the incumbent. It has a proven track record, having been used in more than 90 publications to report data from more than 7000 ancient individuals and to make robust inferences about population history.

The Arbor Complete assay targets the same core set of SNPs as the 1240k, along with an additional valuable set of transversion SNPs. A particular strength of the Arbor assay is that it is commercially available, making it practically available to any researchers who wish to take advantage of the power of whole-genome SNP enrichment. Our implementation of Arbor Complete enrichment did not produce results of as high a quality as the two other assays, but we did not optimize the Arbor protocols in our laboratory as we did for the 1240k and Twist Ancient DNA assays, and thus for this assay, there is the greatest opportunity for improvement relative to the already good performance shown here without optimization.

The Twist Ancient DNA assay was the most efficient of the three in our experiments, capturing sequences overlapping almost all targeted positions with relatively high homogeneity, achieving higher coverage, and having the least allelic bias, making it most easily coanalyzable with shotgun data at nearly all targeted SNPs. Like Arbor Complete, the Twist Ancient DNA assay is commercially available. We have introduced a filter that tags the SNPs most affected by the bias in 1240k enrichment and that provides confidence that Twist data will be robustly coanalyzable with the great majority of ancient human DNA data generated to date.

Because of the multiple advantages associated with the Twist Ancient DNA assay, in June 2021 we performed our last of more than 28,000 1240k captures in our laboratory. Since then, we have enriched more than 9000 libraries with the Twist Ancient DNA assay and published our first data (Fowler et al. 2022). It is important for scientific communities periodically to update their methodologies when there are enough technical improvements, and we believe the advantages of new reagents are now so large that this time has come for ancient human DNA.

## Methods

### DNA extraction and library preparation

We extracted DNA from tooth or bone powder using a manual (Dabney et al. 2013; Korlević et al. 2015) or automated protocol (Rohland et al. 2018) using “Dabney” buffer and silica-coated magnetic beads. We built the extract into indexed single-stranded USER-treated libraries (Gansauge et al. 2020) or into partial-UDG-treated barcoded double-stranded libraries (Rohland et al. 2015). For cleanups after automated library preparation, we used silica-coated magnetic beads and PB (Qiagen), and for cleanups after amplification, we used SPRI beads.

### Target enrichment

The three target enrichment bait reagents all consist of biotinylated DNA probes, and whereas Arbor Complete and 1240k use single-stranded probes (52 bp for 1240k; unknown, to us, for Arbor Complete), Twist Ancient DNA uses double-stranded 80-bp probes. The original protocol for Twist assays (standard protocol for Twist target enrichment for modern DNA pooled libraries) specified one round of enrichment, whereas the protocols for Arbor Complete and 1240k specified two consecutive rounds of enrichment (tailored to ancient DNA libraries enriched in single-plex). Arbor Complete and 1240k had the mitochondrial panel included in our testing (1240k reagent: 3-bp tiled probes of the mitochondrial genome of 52-bp length, spiked in at 0.033%), whereas for Twist Ancient DNA, we only added the Twist Mitochondrial Panel to 19 of the 27 libraries (120-bp probes, spiked in at 1.67%). In our Twist Ancient DNA testing, we added in the mitochondrial DNA probes at a 10th of the concentration we had intended (our plan had been to spike in at 16.7%, but effectively, we used 10× less because the concentration in the kit was 10× lower than expected). In subsequent experiments with the intended concentration, we have obtained more efficient mitochondrial retrieval for Twist than we show in Supplemental Data 6.

For a total of 10 ancient human DNA libraries (five single-stranded and five double-stranded) of varying genomic complexity and endogenous content (Table 1), we enriched for one and, in almost every case, two rounds. Additionally, we enriched 15 high-complexity libraries and two lower-complexity libraries for which we had large amounts of shotgun sequence data to further investigate the performance of each assay. For these libraries, we performed our evaluations based on the recommended number of rounds of enrichment for each assay before the revised recommendations that emerged from this research: one round for Twist Ancient DNA, two rounds for 1240k, and two rounds for Arbor Complete. Both 1240k and Arbor hybridizations were performed manually, and capture and washes were performed using a PerkinElmer EP3 liquid handler. Incubation steps were performed on a thermocycler when different to room temperature. Twist hybridizations, as well as capture and washes, were pipetted using the Agilent Bravo NGS Workstation with a script written by a Twist Bioscience representative. Supplemental Table S4 compares the protocols for the reagents; we present the details in what follows.

### 1240k

Since the development (Fu et al. 2013) of the in-solution enrichment technology that is the basis for the 1240k assay, we have changed the temperature settings in our laboratory's implementation but not buffer composition or volumes. For the experiments reported here, we started with 1 µg of library and hybridized to 1 µg of single-stranded biotinylated bait in a total volume of 34 µL (1 × HI-RPM hybridization buffer [Agilent], 4.4 × Denhardt's solution, 74 ng human Cot-I DNA, 74 ng salmon sperm DNA, 14.5/29 µM each blocking oligos) for at least 16 h at 73°C in a thermocycler. We bound the biotinylated probes to 30 µL MyOne streptavidin C1 beads in binding buffer (1 M sodium chloride, 10 mM Tris-HCl at pH 8.0, 1 mM EDTA at pH8.0, 0.05% Tween-20) for 30 min and washed the beads five times with three different wash buffers (one time for 15 min in WB1: 1× SSC, 0.1% SDS, three stringent washes for 10 min at 57°C each in HWT: 1× GeneAmp PCR Gold Buffer, Applied Biosystems, 0.02% Tween-20; and one time in WB3 [no incubation]: 0.1× SSC, 0.05% Tween-20). We melted the library molecules from the probes with sodium hydroxide, precipitated with ethanol and sodium acetate onto magnetic Sera-Mag SpeedBeads (carboxyl-modified from Cytivia), washed twice with 80% ethanol, eluted in TE, and amplified for 30 cycles using the appropriate primer pairs (depending on whether they were single- or double-stranded libraries) and Herculase II fusion polymerase in a 100 µL total volume. We cleaned up the product with 100 µL 38% SPRI reagent (1:1 ratio) (Fu et al. 2013) and eluted round 1 in 15 µL TE. For round 2, we used 5 µL of the round 1 product (usually 500–700 ng total) and hybridized with 500 ng of single-stranded biotinylated baits again for ∼16 h. The round 2 capture and washes were identical to those of round 1, but we eluted the cleaned PCR product in 50 µL, usually resulting in 50–90 ng/µL product.

### Arbor complete

We used the “myBaits Expert Human Affinities–Complete panel.” The kit was not commercially available at the time of testing, and we therefore used reagents and buffers also used for 1240k as recommended by representatives of Daicel Arbor (see above). We used experimental settings similar to the 1240k settings, with the following adjustments. We hybridized at 70°C and bound to 30 µL MyOne streptavidin C1 beads in binding buffer for 5 min at 70°C. We performed all washes identically to 1240k but performed the three stringent washes at 55°C and reduced the number of amplification cycles to 20 in round 1. We used the entire product in round 2 (except for the 10 libraries for which we tested one and two rounds of capture, where we kept 1/7th for round 1 indexing PCR and sequencing). We performed the final amplification for 12 cycles. The now commercially available kit differs from the settings we used, and the recommended settings can be found online (https://arborbiosci.com/wp-content/uploads/2021/03/myBaits_Expert_HumanAffinities_v1.0_Manual.pdf).

### Twist Ancient DNA

We explored a range of probe lengths, bait reagent volumes, and temperature settings to optimize performance for unmultiplexed low-complexity single-stranded ancient DNA libraries with short insert lengths (four libraries from our set), which is a very different type of enrichment challenge from the one for which Twist protocols were originally designed (multiplexed high-complexity modern libraries with long insert lengths). We started out with the protocol “Protocol_NGS_HybridizationTE_31OCT19_Rev1.” The experimental conditions we identified, which after optimization are substantially different from the protocol optimized by Twist for in-solution enrichment products applied to multiplex modern DNA, are as follows. We used 1 µg of dried library and reconstituted in 7 µL of universal blockers and 5 µL blocker solution. In a second plate, we combined 5 µL of hybridization mix (standard protocol is 20 µL) with 1 µL of Twist Ancient DNA probes (Twist custom probe panel number: TE-94002772; this is an optimized volume based on our testing; the standard protocol from Twist for modern high-quality DNA specifies 4 µL). We melted the (double-stranded) probes for 5 min at 95°C and cooled for 5 min to 4°C. During the 4°C cooling of the probes, we incubated libraries and blockers for 5 min at 95°C. We next equilibrated both plates for 5 min to room temperature. We added the 6 µL of probe (6.167 µL if mitochondrial DNA probes were added) and hybridization buffer to the 12 µL library and blocker, mixed, and overlaid with 30 µL hybridization enhancer and incubated at 62°C (standard is 70°C) in a thermal cycler for at least 16 h. We used 300 µL streptavidin beads (standard is 100 µL) and bound for 30 min at room temperature. In manual processing, we washed beads four times with two different wash buffers; three were stringent washes at 49°C (standard is 48°C). In automated processing, we performed seven washes, of which six were stringent washes at 49°C; the automation protocol is available from Twist Bioscience. We amplified from 50% of the bead slurry with Kapa HiFi HotStart ReadyMix for 23 cycles (standard is fewer cycles depending on target size) with the provided primers (ILMN) for single-stranded libraries or indexing primer for double-stranded libraries in an off-bead PCR. We finally purified the PCRs with 1.8× purification beads (standard is 1×) and eluted in 50 µL TE.

### Sequencing

We sequenced enriched and shotgun libraries on HiSeq X Ten instruments with 2 × 101 cycles and either 2 × 7 cycles (double-stranded libraries) or 2 × 8 cycles (single-stranded libraries) to read the index sequences.

### Data processing

Because the enriched ancient DNA libraries were sequenced in pools, we needed to demultiplex sequences. We did this based on two types of oligonucleotide tags: library-specific barcode pairs (for double-stranded libraries) and index pairs (for all libraries). We merged paired-end sequences requiring a minimum of 15-bp overlap with, at most, one mismatch if base quality was 20 or more or with up to three mismatches of lower base quality. We mapped sequences to the human genome reference (hg19) using *bwa samse* from BWA-v0.6.1 (Li and Durbin 2010). Our choice of reference genome was motivated by this reference genome being the de facto standard in the ancient DNA community. Some sequences will of course align differently to other reference genomes, such as the newer GRCh38. However, in simulations of alignment of sequences flanking targeted SNP positions, only a small proportion of aligned molecules would be expected to map differently. We restricted to merged sequences of at least 30 bp. For analyses in which we were interested in the relative efficiency of the retrieval of molecules at different targeted locations, we measured the coverage before removal of PCR duplicated molecules; for other analyses, we assessed the coverage after the removal of PCR duplicates. To represent each nucleotide position for analyses that required SNP genotype calls, we chose a random sequence at each location, requiring a mapping and base quality of 10 and 20, respectively. Metrics and analyses were computed using the SAMtools (Li et al. 2009) and BCFtools toolkits (Denecek 2021).

### Fraction of published ancient DNA data produced by in-solution enrichment

To compute the proportion of genome-wide ancient human DNA data for which data had been generated by 1240k enrichment (>70%), we used all published data from version v51 of the Allen Ancient DNA Resource (https://reich.hms.harvard.edu/allen-ancient-dna-resource-aadr-downloadable-genotypes-present-day-and-ancient-dna-data), consisting of compiled records of published genome-wide ancient human DNA data as of December 22, 2021.

### Estimated fraction of published ancient human genomes with <10% endogenous DNA

To compute the fraction of individuals with proportions of endogenous DNA below different thresholds, we restricted to published data from our laboratory for which we had at least 15,000 SNPs on Chromosomes 1–22 present targeted by the 1240k assay and for which we had assessed as passing quality control either fully (“PASS”) or with minor concerns (“QUESTIONABLE”). We restricted to individuals for which we had an endogenous DNA proportion estimate for at least one library, and represented each individual by the library with the most endogenous DNA.

## Data access

All processed sequencing data generated in this study have been submitted to the European Nucleotide Archive (ENA; https://www.ebi.ac.uk/ena/browser/home) under accession number PRJEB54983.

## Supplementary Material

Supplemental Material
