# Supplementary material for: Three assays for in-solution enrichment of ancient human DNA at more than a million SNPs
Source: Genome Res. 2022 Nov-Dec;32(11-12):2068–78. doi: 10.1101/gr.276728.122 (PMC9808625; doi:10.1101/gr.276728.122)
Supplement: Supplemental Material [file supp_32_11-12_2068__DC1.html]

Three assays for in-solution enrichment of ancient human DNA at more than a million SNPs — Supplemental Material 

# Three assays for in-solution enrichment of ancient human DNA at more than a million SNPs

## Supplemental Material

- Supplemental\_Material.pdf
- Supplemental\_Data\_1\_twistSNP\_1352529bp.txt.zip
- Supplemental\_Data\_2\_methylation\_80000bp.txt.zip
- Supplemental\_Data\_3\_HARS\_857339bp.txt.zip
- Supplemental\_Data\_4\_resequencing\_2577bp.txt.zip
- Supplemental\_Data\_5\_Ycoverage\_10446037bp.txt.zip
- Supplemental\_Data\_6\_mtDNA\_16569bp.txt.zip
- Supplemental\_Data\_7\_chr1.txt.gz
- Supplemental\_Data\_7\_chr2.txt.gz
- Supplemental\_Data\_7\_chr3.txt.gz
- Supplemental\_Data\_7\_chr4.txt.gz
- Supplemental\_Data\_7\_chr5.txt.gz
- Supplemental\_Data\_7\_chr6.txt.gz
- Supplemental\_Data\_7\_chr7.txt.gz
- Supplemental\_Data\_7\_chr8.txt.gz
- Supplemental\_Data\_7\_chr9.txt.gz
- Supplemental\_Data\_7\_chr10.txt.gz
- Supplemental\_Data\_7\_chr11.txt.gz
- Supplemental\_Data\_7\_chr12.txt.gz
- Supplemental\_Data\_7\_chr13.txt.gz
- Supplemental\_Data\_7\_chr14.txt.gz
- Supplemental\_Data\_7\_chr15.txt.gz
- Supplemental\_Data\_7\_chr16.txt.gz
- Supplemental\_Data\_7\_chr17.txt.gz
- Supplemental\_Data\_7\_chr18.txt.gz
- Supplemental\_Data\_7\_chr19.txt.gz
- Supplemental\_Data\_7\_chr20.txt.gz
- Supplemental\_Data\_7\_chr21.txt.gz
- Supplemental\_Data\_7\_chr22.txt.gz
- Supplemental\_Data\_7\_chrX.txt.gz
- Supplemental\_Data\_7\_chrY.txt.gz
