# Supplementary material for: Three assays for in-solution enrichment of ancient human DNA at more than a million SNPs
Source: Genome Res. 2022 Nov-Dec;32(11-12):2068–78. doi: 10.1101/gr.276728.122 (PMC9808625; doi:10.1101/gr.276728.122)

Supplemental Text S1: Content in the Twist Ancient DNA bait set beyond 1240k

Here we discuss the procedure used to identify a set of bait sequences to submit for design. Twist BioSciences performed the final design, which involved determining the relative concentrations of different oligonucleotides using a proprietary algorithm unknown to the authors.

(1a) Adding 94,586 polymorphisms on Chromosomes 1-22 and X

For the Twist Ancient DNA reagent, we began by attempting to bait all 1,233,013 SNPs on the 1240k reagent. We then added additional content to target SNPs of phenotypic significance or SNPs improving characterization of variation on the Y Chromosome.

- *"GWAS" SNPs (SNPs associated with phenotypes in Genome-Wide Association Studies)*
  We used a list of 236,638 SNPs that are genome-wide significant in one of 4,155 GWAS's on 558 traits in a diverse set of populations (Watanabe et al. 2019). In contrast to the GWAS catalog database (Welter et al. 2014), this only includes SNPs from GWAS of at least 50,000 individuals.

- *"RELATE" SNPs*
  We included SNPs estimated to have been under recent selection in any of 26 diverse modern populations from the 1000 Genomes Project (The 1000 Genomes Project Consortium 2015) based on distortions in coalescent tree shapes (Speidel et al. 2019). We selected 61,308 SNPs with p-values < 10^{-5} in any population.

- *"Clinvar" SNPs*
  We included 32,689 SNPs from the Clinvar database by selecting all variants where the highest reported allele frequency is >1% (Landrum et al. 2020) (https://www.ncbi.nlm.nih.gov/clinvar/). These SNPs are highly enriched for coding, non-synonymous variants.

- *"Polyfun" SNPs*
  We included 75,592 fine-mapped SNPs falling in regions with functional annotations that are enriched for heritability for a range of complex traits, specifically all SNPs with Posterior Inclusion Probability of >0.1 (Weissbrod et al. 2020).

(b) Linkage disequilibrium (LD) pruning to remove genetically correlated SNPs

We pruned the selected SNPs for linkage disequilibrium in 2,261 individuals from the 1000 Genomes Project. For pruning, we use the PLINK (Purcell et al. 2007) command --indep-pairwise 1000 100 0.9.

We computed LD for each of the remaining SNPs to the core set of 1240k SNPs using the command --r2 --ld-window-r2 0.2 --ld-window 10 --ld-window-kb 1000. We excluded all SNPs with LD greater than 0.9 to any 1240k SNP.

(c) Quality control

We characterized SNPs from all sources by their dbSNP reference numbers (rs-IDs) as well as their reference and variant alleles. We filtered out insertion/deletion polymorphisms. We mapped rs-IDs to

chromosome and position and determined alleles using the Ensembl database for genome build GRCh37 (hg19), accessed through biomaRt (http://www.biomart.org/). We used the *hg19* reference sequence ("hg19_1000g.fa.gz") to obtain 52 bp flanking either side. For multi-allelic sites, we kept two variants identified in the original sources. We designated the allele in the *hg19* reference sequence as "ref", and the alternative allele as "alt".

Supplemental Text 1 Table A shows a record of the SNPs deriving from each of these four methodologies, including the number retained after the different pruning steps; this identified 94,586 SNPs. Supplemental Text 1 Table B shows the distribution across chromosomes for each method.

Supplemental Text 1 Table A: SNPs selected from each source (some overlap, so total is not sum)

Name	Initial	Not in 1240k	After pruning	R^2<0.9	Would keep	Mean allele frequency	Mean R^2 (>0.2)	Mean R^2 (≤0.2)
Clinvar	32705	27495	20544	17262	17601	0.167	0.7	0.337
GWAS	236638	160819	66857	38540	38478	0.401	0.79	0.012
Polyfun	75592	59500	42088	32430	33145	0.279	0.72	0.174
Relate	61308	49701	23228	14579	14428	0.419	0.78	0.008
Total	375408	276824	140520	93812	94586	0.361	0.77	0.066

Note: "Would keep" includes SNPs not in the 1000 Genomes Project, excluding SNPs with mismatching alleles or positions.

Supplemental Text 1 Table B: Number of newly targeted SNPs by chromosome

Chromosome	Clinvar	GWAS	Polyfun	Relate
1	1496	2932	3053	1052
2	1612	4291	2773	1348
3	890	2775	2094	989
4	693	1902	1497	922
5	922	2486	1826	830
6	908	3020	1903	764
7	741	2030	1883	800
8	668	2300	1279	820
9	907	2158	1391	907
10	684	1549	1479	702
11	1031	2066	1677	714
12	888	2019	1841	644
13	392	999	909	458
14	533	1036	910	527
15	613	1517	1149	476
16	1042	1081	1433	713
17	1095	1156	1646	380
18	341	785	771	379
19	936	607	1557	307
20	464	1204	1098	301
21	324	80	410	192
22	378	485	567	203
X	43	NA	NA	NA
Total	17601	38478	33146	14428

Supplemental Text Figure A shows the allele frequency distribution of the variant allele. Supplemental Text Figure B shows the distribution of maximum R^2 to any 1000 Genomes Project SNPs.

Supplemental Text Figure A: Allele frequency distribution by source of newly added SNPs

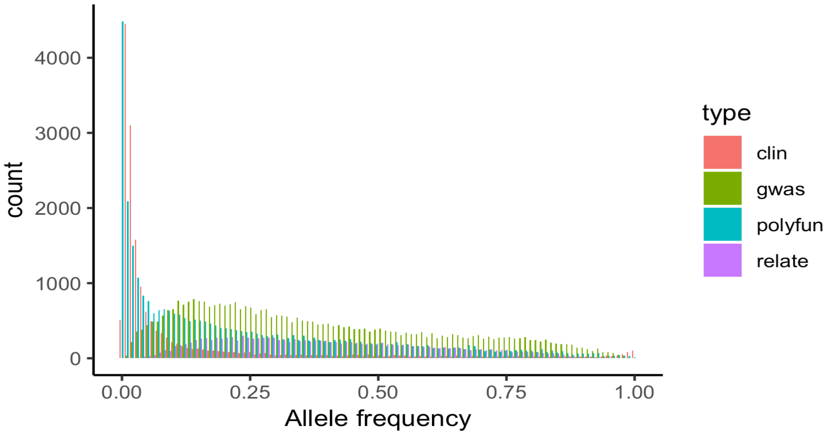

Supplemental Text Figure B: Linkage disequilibrium distribution by source.

We characterize each SNP according to its highest R^2 to any 1240k target SNP. The histogram excludes all SNPs with $R^2>0.9$, and sets the LD for all SNPs with LD<0.2 to 0.

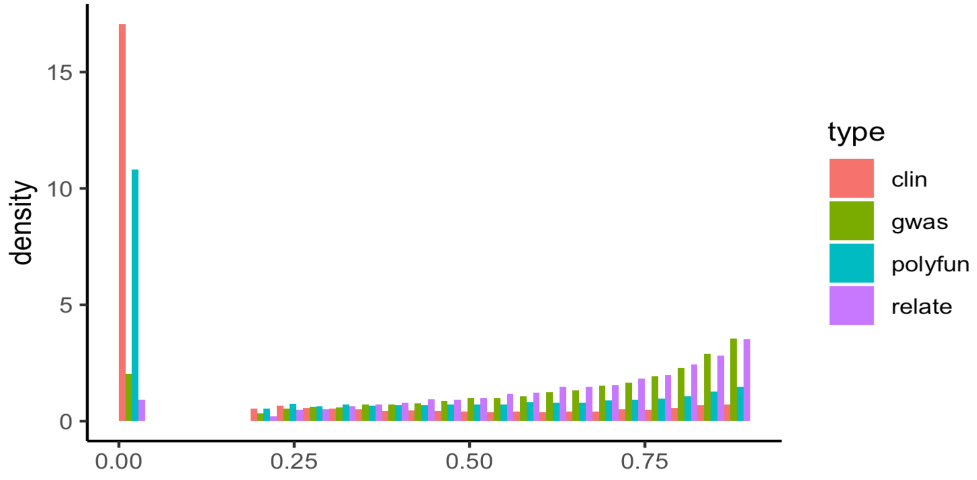

Finally, we manually added 15 phenotypically important multi-allelic polymorphisms and 6 insertion/deletion targets where we tiled both alternative alleles (Supplemental Text Table C).

Supplemental Text Table C: Manual addition of 15 multiallelic SNPs and 6 insertion/deletions

**Target**	**Chr**	**Ascer-tainment**	**Target type**	**Position of site in hg19 (start for Indel)**	**Beginning of targeted sequence in hg19**	**End of targeted sequence in hg19**	**Ref**	**Var(s)**	**Tiled Oligo-nucleotide**
rs77931234	1	Medium-chain acyl-CoA dehydrogenase deficiency	Multiallelic position (design reference)	76226846	76226794	76226898	A	C,G,T	TTTTTAATTCTAGCACCAAGCAATATCATTTATGCTGGCTGAAATGGCAATGTAAGTTGAACTAGCTAGAATGAGTTACCAGAGAGCAGCTTGGGAGGTTGATTC

snp_2_136608745	2	lactase persistence	Multiallelic position (design reference)	136608745	136608693	136608797	A	C,T	TTGTAGGGTCTAAGTACATTTTTCCTGAATGAAAGGTATTAAATGGTAACTTTCGTCTTTATGCACTCTATAAACTATGACGTGATCGTCTCCGTCTAACAACTA
rs75030631	5	Spinal Muscular Atrophy	Multiallelic position (design reference)	70220935	70220883	70220987	C	G,A	ACTCTTAAGAAGGGACGGGGCCCCACGCTGCGCACCCGCGGGTTTGCTATGGAGATGAGCAGCGGCGGCAGTGGTGGCGGCGTCCCGGAGCAGGAGGATTCCGTG
rs1800562	6	Hereditary Hemochromatosis	Multiallelic position (design reference)	26093141	26093089	26093193	G	A,T	CAGGGCTGGATAACCTTGGCTGTACCCCCTGGGGAAGAGCAGAGATATACGTTCCAGGTGGAGCACCCAGGCCTGGATCAGCCCCTCATTGTGATCTGGGGTATG
rs111033171	9	Familial Dysautonomia	Multiallelic position (design reference)	111662096	111662044	111662148	A	G,T	ATTGTCTTCACACATAAATCACAAGCTAACTAGTCGCAAACAGTACAATGGCTCTTACTTGTCCAACCACTTCCGAATCTGAGCTAAAACCAGGGCTCGATGATG
rs33985472	11	β-Thalassemia	Multiallelic position (design reference)	5246715	5246663	5246767	T	C,G	TAAAATATTCAGAAATAATTTAAATACATCATTGCAATGAAAATAAATGTTTGTTATTAGGCAGAATCCAGATGCTCAAGGCCCTTCATAATATCCCCCAGTTTA
rs35004220	11	β-Thalassemia	Multiallelic position (design reference)	5248050	5247998	5248102	C	T,A	ACCTCTGGGTCCAAGGGTAGACCACCAGCAGCCTAAGGGTGGGAAAATAGACAAATAGGCAGAGAGAGTCAGTGCCTATCAGAAACCCAAGAGTCTTCTCTGTCT
rs80338863	11	Smith-Lemli-Opitz syndrome	Multiallelic position (design reference)	71148990	71148938	71149042	C	G,T	TGGTCTTCAGGTACCAGGTTTCGTTCCAGAAGAAGTCAATCACGTAGATGGCTTGCAAGACAGAAGCAGCCGCTGACCACCCCCGGCCCTCCTGGGGCCCCCATG
rs5030858	12	Phenylketonuria	Multiallelic position (design reference)	103234271	103234219	103234323	G	A,C	TCCAAGACCTCAATCCTTTGGGTGTATGGGTCGTAGCGAACTGAGAAGGGCCCAGGTATTGTGGCAGCAAAGTTCCTAAGACCAAAACCACAGGCTTGAGTGAAG
snp_15_28496195	15	pigmentation	Multiallelic position (design reference)	28496195	28496143	28496247	A	G,C	ATGTCCCATACAGGACCCCACGTGCCACAGGAACCAAAAAGTCACATGCAGCCAGGATGAAGACACAGGAGACAACCTGTGTGGACAGCACAGAGCCACCTGCCG
snp_16_89383725	16	pigmentation	Multiallelic position (design reference)	89383725	89383673	89383777	T	C,G	ACAGGAATGGCAGCTTTGAGCAGGAAGGAGAACAGAGAAGGGTCAAGCACTTGGTAGTGGCAGAAAGGGACGCATGGCCTAGGGTGTGGCTGTGTTCTGGGTGGC
rs3212355	16	pigmentation	Multiallelic position (design reference)	89984378	89984326	89984430	C	T,G	GAGTGAACCCAGGAAGATGCCTGCAGTGGGTGCCAGGGCCCCTCTCCACCGTGCCTGCTGGGCTTCGGGGCCACGCCCGACTGCTGTGAACGGCCTGCGGAGCAC
snp_16_89986122	16	pigmentation	Multiallelic position (design reference)	89986122	89986070	89986174	C	A,T	TGGGCGCCATCGCCGTGGACCGCTACATCTCCATCTTCTACGCACTGCGCTATCACAGCATCGTGACCCTGCCGCGGGCGCGGCGAGCCGTTGCGGCCATCTGGG
snp_16_90024206	16	pigmentation	Multiallelic position (design reference)	90024206	90024154	90024258	A	G,T	CTCTCTCAGGCGGTGGTCTCTCTCTCGGCCTCAGGGGCTGAGGTAGAAGGGCTCGAGACAGGCAGGGTGGAAGACGGGCCCTCACCCCACTGCGGGAGGTTTCCC

snp_20_32665748	20	pigmentation	Multiallelic position (design reference)	32665748	32665696	32665800	A	G,T	GTTCCCACATTTTACCCTGTGAGGAAATCGAGGCTCAGAAAGGCTGAGTGGCTTGCTCAGGGCATCAGCTCGTAGGGACTGAGCCAGGGTTGGAGTCCAGACTGA
rs333	3	HIV-AIDS immunity	Insertion/deletion (design both versions)	46414947	46414908	46415012	GTCAGTATCAATTCTGGAAGAATTTCCAGACA	deletion	AAGGTCTTCATTACACCTGCAGCTCTCATTTTCCATACAGTCAGTATCAATTCTGGAAGAATTTCCAGACATTAAAGATAGTCATCTTGGGGCTGGTCCTGCCGC
rs333.deletion	3	HIV-AIDS immunity	Insertion/deletion (design both versions)	46414947	46414893	46415029	GTCAGTATCAATTCTGGAAGAATTTCCAGACA	deletion	CCAGATCTCAAAAAGAAGGTCTTCATTACACCTGCAGCTCTCATTTTCCATACATTAAAGATAGTCATCTTGGGGCTGGTCCTGCCGCTGCTTGTCATGGTCATC
rs113993960	7	Cystic Fibrosis	Insertion/deletion (design both versions)	117199646	117199594	117199698	CTT	deletion	TCTGTTCTCAGTTTTCCTGGATTATGCCTGGCACCATTAAAGAAAATATCATCTTTGGTGTTTCCTATGATGAATATAGATACAGAAGCGTCATCAAAGCATGCC
rs113993960.deletion	7	Cystic Fibrosis	Insertion/deletion (design both versions)	117199646	117199593	117199700	CTT	deletion	TTCTGTTCTCAGTTTTCCTGGATTATGCCTGGCACCATTAAAGAAAATATCATTGGTGTTTCCTATGATGAATATAGATACAGAAGCGTCATCAAAGCATGCCAA
rs387906309	15	Tay-Sachs	Insertion/deletion (design both versions)	72638921	72638870	72638974	insertion	GATA	TCAAATGCCAGGGGTTCCACTATGTAGAAATCCTTCCAGTCAGGGCCATAGGATATACGGTTCAGGTACCAGGGGGCAGAGAGAAGGGCCCGGAAGCCGGCCTTG
rs387906309.insertion	15	Tay-Sachs	Insertion/deletion (design both versions)	72638921	72638872	72638972	insertion	GATA	AAATGCCAGGGGTTCCACTATGTAGAAATCCTTCCAGTCAGGGCCATAGGATAGATATACGGTTCAGGTACCAGGGGGCAGAGAGAAGGGCCCGGAAGCCGGCCT
rs41474145	16	α-Thalassemia	Insertion/deletion (design both versions)	223008	222956	223060	TGAGG	deletion	GGGTAAGGTCGGCGCGCACGCTGGCGAGTATGGTGCGGAGGCCCTGGAGAGGTGAGGCTCCCTCCCCTGCTCCGACCCGGGCTCCTCGCCCGCCCGGACCCACAG
rs41474145.deletion	16	α-Thalassemia	Insertion/deletion (design both versions)	223008	222953	223062	TGAGG	deletion	CTGGGGTAAGGTCGGCGCGCACGCTGGCGAGTATGGTGCGGAGGCCCTGGAGAGGCTCCCTCCCCTGCTCCGACCCGGGCTCCTCGCCCGCCCGGACCCACAGGC
rs63751471	16	α-Thalassemia	Insertion/deletion (design both versions)	223510	223463	223567	CTCCCCGCCGAG	deletion	CTGCACAGCTCCTAAGCCACTGCCTGCTGGTGACCCTGGCCGCCCACCTCCCCGCCGAGTTCACCCCTGCGGTGCACGCCTCCCTGGACAAGTTCCTGGCTTCTG
rs63751471.deletion	16	α-Thalassemia	Insertion/deletion (design both versions)	223510	223463	223579	CTCCCCGCCGAG	deletion	CTGCACAGCTCCTAAGCCACTGCCTGCTGGTGACCCTGGCCGCCCACTTCACCCCTGCGGTGCACGCCTCCCTGGACAAGTTCCTGGCTTCTGTGAGCACCGTGC
rs587776730	X	Favism	Insertion/deletion (design both versions)	153761232	153761189	153761293	C	deletion	ACGGCTGCAAAAGTGGCGGTGGTGGACCCGCGGGGCACCGTGGGGTCGTCCAGGTACCCTTTGGTGGCCTCGCCCTCTCCATCGGGGTTCCCCACGTACTGGCCC

rs587776730.deletion	X	Favism	Insertion/deletion (design both versions)	153761232	153761177	153761305	C	deletion	ACATAGAGGACGACGGCTGCAAAAGTGGCGGTGGTGGACCCGCGGGGCACCGTGGCCTCGCCCTCTCCATCGGGGTTCCCCACGTACTGGCCCAGGACCACATTG

(1b) Targeting 81,925 polymorphisms on chromosome Y

To identify Y chromosome targets, we started with 32,670 chromosome Y SNPs from the 1240k reagent. These had been identified by starting with ISOGG 9.77 SNPs (https://isogg.org/), and then merging with SNPs identified as polymorphic in the Simons Genome Diversity Project (Fu et al. 2015; Mallick et al. 2016).

For our redesign, we added in 69,991 Y SNPs from the ISOGG Y SNP index version 14.199 downloaded Nov. 5 (https://isogg.org/). We started with 88,795 polymorphisms in the download, removed ones with duplicate positions, and restricted to SNPs that are biallelic for the alleles A/C/G/T.

After merging and removing duplicates, this generated 88,023 SNPs. We removed SNPs monomorphic in the existing 1240k enrichment dataset, or that had coverage counts of <10%, leaving 81,925 SNPs.

In contrast to the 94,586 SNPs identified in Section 1a, which represent a supplement to the 1240k content on chromosomes 1-22 and X, for the Y chromosome the 81,925 SNPs we targeted are a replacement of the 1240k content on chromosome Y.

(1c) Final count of SNPs

The total number of SNPs targeted for the reagent is:

1,200,343	1240k content on chromosomes 1-22 and Y
94,586	Newly designed phenotypic discussed in Section 1a
81,925	Fully redesigned Y chromosome content discussed in Section 1b
1,376,854	Total

For each targeted SNP, we randomly selected a third allele to represent each position and flanked it 52 bp on either side according to the h*g19* genome sequence. We then mapped to *hg19*. Our choice of mapping to this reference, even though there are a number of newer human reference genomes available was motivated by this genome as the standard in the ancient DNA community.. One caveat is that some reads might align differently to newer references such as GRCh38, however in separate analyses (not shown) based on the alignments of pseudo-molecules based on SNP positions, only a small number of molecules would be affected.

After removing oligonucleotides that mapped unreliably with a score of MAPQ<23, or that mapped to a location that disagreed with the recorded positions, or that was duplicated in its sequence compared to another in the dataset, or that failed other quality controls, our design file targeted 1,352,535 SNPs.

(1d) Tiled regions (with either 1× or 2× tiling)

Beyond SNP targeting, we also added in probes to bait additional genomic regions.

- *"Methylation" targets*
  We are grateful to Steve Horvath and Vagheesh Narasimhan for providing us with the coordinates of 40,000 CpG dinucleotides chosen to be locations where methylation rates are correlated to the skeletally determined ages of ancient individuals. These CpG dinucleotides are also ones where methylation rates have been shown to be well-correlated to the ages of living individuals. Of these targets, we successfully designed single probes for 39,886 (we did not design probes for the others due to repetitive flanking sequence).

- *"Human Accelerated Region (HAR)" targets*
  We are grateful to Ryan Doan for sharing with us a list of 3,171 Human Accelerated Regions (HARs) spanning 857,339 nucleotides (Girskis et al. 2021). We tiled each of these regions twice (with 80bp probes overlapping every 40bp).

- *"Gene resequencing" targets*
  This includes 9 contiguous regions in 3 genes, specified in hg19 coordinates. The segments target SNPs relevant to β-thalassemia (chr. 11: 5247022-5247193 and 5248114-5248429), α-thalassemia (chr. 16: 222873-223052 and 223469-223733), and favism (chr. X: 153220145-153220335, 153760378-153761377, 153761761-153761889, 153763362-153763532, 153764171-153764423, and 153774226-153774316). The SNPs are rs34690599, rs34451549, rs35724775, rs33915217, rs33971440, rs33960103, rs33986703, rs34716011, rs63750783 , rs334, rs34598529, rs33944208, rs111033603, rs281864819, rs41474145, rs63750404, rs63751471, rs33987053, rs41397847, rs41464951, rs63751269, rs137852348, rs137852344, rs72554664, rs72554665, rs72554665, rs137852324, rs137852317, rs137852337, rs2230037, rs137852336, rs137852323, rs137852335, rs137852316, rs137852316, rs137852321, rs137852334, rs137852320, rs137852322, rs2230036, rs387906468, rs137852329, rs137852345, rs137852333, rs137852342, rs5030869, rs587776730, rs76723693, rs137852347, rs137852339, rs137852327, rs74575103, rs137852318, rs137852346, rs137852328, rs137852328, rs137852319, rs137852326, rs137852332, rs137852332, rs137852330, rs5030868, rs267606836, rs5030872, rs5030872, rs137852343, rs137852331, rs137852314, rs2515904, rs137852313, rs137852341, rs1050829, rs137852349, rs1050828, rs137852315, rs76645461, and rs78478128. We tiled segments with 80bp probes staggered every 40bp.

Literature Cited for Supplemental Text 1

Fu Q, Hajdinjak M, Moldovan OT, Constantin S, Mallick S, Skoglund P, Patterson N, Rohland N, Lazaridis I, Nickel B et al. 2015. An early modern human from Romania with a recent Neanderthal ancestor. *Nature* **524**: 216-219.

Girskis KM, Stergachis AB, DeGennaro EM, Doan RN, Qian X, Johnson MB, Wang PP, Sejourne GM, Nagy MA, Pollina EA et al. 2021. Rewiring of human neurodevelopmental gene regulatory programs by human accelerated regions. *Neuron* **109**: 3239-3251 e3237.

Landrum MJ, Chitipiralla S, Brown GR, Chen C, Gu B, Hart J, Hoffman D, Jang W, Kaur K, Liu C et al. 2020. ClinVar: improvements to accessing data. *Nucleic acids research* **48**: D835-D844.

Mallick S, Li H, Lipson M, Mathieson I, Gymrek M, Racimo F, Zhao M, Chennagiri N, Nordenfelt S, Tandon A et al. 2016. The Simons Genome Diversity Project: 300 genomes from 142 diverse populations. *Nature* **538**: 201-206.

Purcell S, Neale B, Todd-Brown K, Thomas L, Ferreira MA, Bender D, Maller J, Sklar P, de Bakker PI, Daly MJ et al. 2007. PLINK: a tool set for whole-genome association and population-based linkage analyses. *Am J Hum Genet* **81**: 559-575.

Speidel L, Forest M, Shi S, Myers SR. 2019. A method for genome-wide genealogy estimation for thousands of samples. *Nat Genet* **51**: 1321-1329.

The 1000 Genomes Project Consortium. 2015. A global reference for human genetic variation. *Nature* **526**: 68-74.

Watanabe K, Stringer S, Frei O, Umicevic Mirkov M, de Leeuw C, Polderman TJC, van der Sluis S, Andreassen OA, Neale BM, Posthuma D. 2019. A global overview of pleiotropy and genetic architecture in complex traits. *Nat Genet* **51**: 1339-1348.

Weissbrod O, Hormozdiari F, Benner C, Cui R, Ulirsch J, Gazal S, Schoech AP, van de Geijn B, Reshef Y, Marquez-Luna C et al. 2020. Functionally informed fine-mapping and polygenic localization of complex trait heritability. *Nat Genet* **52**: 1355-1363.

Welter D, MacArthur J, Morales J, Burdett T, Hall P, Junkins H, Klemm A, Flicek P, Manolio T, Hindorff L et al. 2014. The NHGRI GWAS Catalog, a curated resource of SNP-trait associations. *Nucleic acids research* **42**: D1001-1006.

Supplemental Text S2: EM Algorithm to Correct for Binomial Sampling Variance

We have empirical counts of reference and variant alleles for large numbers of probable heterozygous positions. We describe our algorithm to deconvolve the noise to learn the underlying distribution of reference bias.

We consider a set of reference and variant counts (typically summing to 100 or more). At SNP *k* we observe a_k reference and b_k variant alleles. We suppose the 'true' allele frequency of reference is $z_k = z$ which we can think of as the frequency we would observe if the coverage were infinite. We wish to learn the probability distribution of z. We ignore the case that the observed counts are not polymorphic, so we assume $a_k, b_k \geq 1$.

Let us model z_k as lying on a mesh; for instance, $z_k = i/100$ for some i = 1…99. We propose to estimate $p_i = (z_k = i/100)$. Write α_i = i/100; β_i = (100-i)/100. The log likelihood of our observation for SNP *k* is:

$$\mathcal{L}(k) = \log\left(\sum_i \alpha_i^{a_k}\,\beta_i^{b_k} + (a_k + b_k)log2\right)$$

The last term is not essential, but good technique is to score against some random model; here that a_k is from a fair coin toss (50% probability heads). The overall log likelihood is:

$$\mathcal{L} = \mathcal{L}(\mathbf{p}) = \sum_i \mathcal{L}(k)$$

$\mathcal{L}$ is easily maximized by an EM algorithm. Write:

$$l(i,k) = \log p_i + a_k \log\alpha_i + b_k \log\beta_i$$

$$lmax_k = \max_i l(i,k)$$

$$\theta(i,k) = \exp(l(i,k) - lmax_k)$$

$$\gamma(i,k) = \frac{\theta(i,k)}{\sum_j \theta(j,k)}$$

Thus, $\gamma(i,k)$ is the posterior probability that $z_k = \alpha_i$. Re-estimates are now simply:

$$\widehat{p_i} = \sum_k \gamma(i,k)/N$$

where N is the number of SNPs. Standard EM shows that:

$$\mathcal{L}(\widehat{\mathbf{p}}) \geq \mathcal{L}(\mathbf{p})$$

We iterate until convergence. We implemented this in C to produce the inferences in Figure 4.

Supplemental Table S1: Sequencing results for all 27 libraries

A total of 10 libraries were sequenced after both the first and second round of enrichment (except for S1633.E1.L1 and S10871.E1.L6 which were not enriched after a second Twist round). The bottom 17 libraries reflect 2, 2 and 1 rounds of enrichment for 1240k, Arbor and Twist respectively. DS - double-stranded, SS - single-stranded.

			**Merged reads prior to removal of PCR duplicates**				**Mean length of merged reads**				**Percentage of merged reads overlapping core set of 1,150,639 autosomal SNPs prior to removal of PCR duplicates (this does not include sequences that land close to but not overlapping the targets, or sequences successfully enriched for targets outside the core set)**			**Number of the core set of 1,150,639 autosomal SNPs covered at least once**				**Mean coverage after duplicate removal on core set of autosomal SNPs (unique sequences overlapping the 1,150,639 autosomal SNPs targeted by all three reagents, divided by number of targets)**			
**Library ID**	**lib. type**	**% aligning to human in shotgun sequencing**	**Shotgun**	**1240k**	**Arbor**	**Twist**	**Shot.**	**1240k**	**Arbor**	**Twist**	**1240k**	**Arbor**	**Twist**	**Shotgun**	**1240k**	**Arbor**	**Twist**	**Shotgun**	**1240k**	**Arbor**	**Twist**
**10 library set - 1 round of enrichment for all data types except shotgun**																					
S20720.Y1.E1.L1	DS	0.10%	251,053	95,278,044	119,451,860	178,421,670	44	48	46	43	3.17%	0.27%	0.60%	35	4,010	3,826	4,351	0.000030	0.004	0.003	0.004
S20721.Y1.E1.L1	DS	1.2%	156,117	91,159,969	97,037,453	104,752,984	44	47	47	47	8.9%	2.4%	6.0%	159	38,937	37,271	41,516	0.000133	0.036	0.033	0.039
S21299.Y1.E1.L1	DS	2.0%	48,278	102,561,843	83,445,818	229,480,365	53	61	61	56	15.4%	5.8%	11.4%	47	373,893	311,833	419,480	0.000041	0.425	0.328	0.493
S20703.Y1.E1.L1	DS	6.6%	219,514	92,428,434	94,887,900	204,399,952	58	66	67	63	16.0%	9.2%	25.9%	584	773,139	725,363	916,293	0.000489	1.290	5.826	1.757
S1633.E1.L1	DS	86.7%	2,727,670,965	83,318,054	100,160,597	176,327,313	44	53	53	50	19.2%	9.6%	31.9%	1,147,352	994,422	1,025,646	1,125,216	27.572747	9.528	5.826	27.187
S8432.E1.L9	SS	0.17%	65,834	72,216,321	49,468,219	240,555,004	42	40	40	36	0.32%	0.09%	0.15%	5	9,980	7,414	18,747	0.000004	0.009	0.006	0.017
S2818.Y1.E4.L1	SS	1.2%	70,741	51,539,481	49,474,365	188,783,289	53	44	43	40	2.1%	0.59%	0.52%	191	18,937	19,906	30,797	0.000160	0.017	0.018	0.028
S13982.Y1.E8.L1	SS	6.9%	70,180	47,411,908	37,978,331	111,587,418	38	40	40	37	8.9%	2.4%	5.8%	63	99,090	94,349	168,002	0.000054	0.092	0.087	0.164
S10872.E1.L4	SS	4.2%	1,862,592	63,248,591	42,084,693	395,280,379	51	58	50	48	8.6%	0.50%	8.3%	1,755	766,012	145,853	1,108,683	0.001506	1.742	0.148	5.109
S10871.E1.L6	SS	42.2%	531,724,501	63,585,236	55,434,463	194,734,351	49	53	54	48	12.8%	8.3%	24.1%	1,123,329	984,211	874,574	1,132,162	4.050870	5.635	2.995	22.876
**10 library set - 2 rounds of enrichment for all data types except shotgun**																					
S20720.Y1.E1.L1	DS	0.10%	251,053	154,968,445	50,881,006	120,715,793	44	50	48	44	18.0%	4.4%	4.0%	35	4,046	3,567	4,270	0.000030	0.006	0.003	0.005
S20721.Y1.E1.L1	DS	1.2%	156,117	138,240,603	105,047,509	93,769,358	44	48	48	49	29.9%	11.9%	19.6%	159	38,877	36,345	40,495	0.000133	0.040	0.034	0.042
S21299.Y1.E1.L1	DS	2.0%	48,278	150,336,633	108,058,253	103,616,402	53	62	62	60	41.3%	24.2%	29.0%	47	376,547	316,466	404,683	0.000041	0.444	0.340	0.478
S20703.Y1.E1.L1	DS	6.6%	219,514	255,052,779	111,154,612	90,643,234	58	66	66	65	42.3%	23.3%	40.6%	584	817,446	692,005	877,949	0.000489	1.481	1.057	1.605
S1633.E1.L1*	DS	86.7%	2,727,670,965	393,161,016	94,405,383	NA	44	55	55	n/a	38.7%	22.7%	n/a	1,147,352	1,065,225	942,538	n/a	27.572747	26.654	6.664	n/a
S8432.E1.L9	SS	0.17%	65,834	32,205,778	41,587,887	104,852,445	42	42	42	43	13.2%	3.4%	2.2%	5	9,839	8,116	15,901	0.000004	0.009	0.007	0.015
S2818.Y1.E4.L1	SS	1.2%	70,741	52,678,133	63,282,613	110,858,903	53	45	44	44	23.4%	14.9%	7.7%	191	18,870	20,663	25,591	0.000160	0.019	0.020	0.026
S13982.Y1.E8.L1	SS	6.9%	70,180	49,807,292	59,662,915	25,380,559	38	41	40	40	32.9%	23.9%	22.7%	63	91,750	94,644	104,093	0.000054	0.088	0.091	0.099
S10872.E1.L4	SS	4.2%	1,862,592	150,903,215	61,320,864	83,020,755	51	60	61	50	36.5%	16.4%	31.0%	1,755	863,816	534,501	1,057,659	0.001506	2.469	1.120	3.995
S10871.E1.L6*	SS	42.2%	531,724,501	271,351,127	65,680,438	NA	49	57	59	n/a	37.4%	28.0%	n/a	1,123,329	1,080,929	863,274	n/a	4.050870	21.284	5.728	n/a
**17 library set - 2 rounds of enrichment for 1240k, 2 rounds of enrichment for Arbor Complete, 1 round of enrichment for Twist Ancient DNA**																					
S2949.E1.L7	DS	1.7%	355,389,471	115,165,304	104,071,862	121,477,955	45	46	47	52	20.2%	3.2%	11.2%	9,157	8,233	8,404	8,305	0.007933	0.011	0.008	0.012
S11857.E1.L1	DS	7.5%	325,565,070	104,040,047	97,458,534	122,812,661	43	44	44	48	25.9%	7.0%	21.3%	36,112	30,035	32,008	31,342	0.031811	0.034	0.030	0.039
S10871.E1.L1	DS	52.6%	3,392,817,802	121,068,282	116,546,266	86,963,332	43	53	50	45	42.7%	25.7%	27.3%	1,099,029	864,395	861,995	1,000,935	5.291361	3.324	2.555	3.846
S1734.E1.L1	DS	73.9%	2,659,971,741	119,325,041	102,138,788	114,955,866	47	54	56	51	33.5%	23.6%	32.2%	1,148,681	988,673	975,842	1,128,780	24.002465	14.997	7.888	21.993
S1583.E1.L1	DS	68.7%	3,389,551,748	111,077,550	105,916,375	114,884,025	43	55	55	51	40.0%	23.7%	29.3%	1,144,814	955,084	955,462	1,112,846	28.168891	15.903	7.888	20.676
S5950.E1.L1	DS	69.6%	3,134,086,352	104,660,609	106,370,574	100,976,181	44	58	61	55	40.8%	24.3%	32.9%	1,149,674	960,933	983,961	1,127,994	29.167912	16.330	9.070	21.185
S4795.E1.L1	DS	79.3%	2,139,845,680	122,810,057	102,313,347	75,602,282	50	58	58	52	39.5%	19.7%	30.9%	1,149,061	991,301	960,201	1,115,350	24.278570	17.828	7.643	15.476
S1965.E1.L1	DS	78.3%	2,629,697,020	109,876,861	109,704,294	119,062,251	45	56	56	51	42.9%	24.3%	31.7%	1,148,250	976,230	984,875	1,125,607	26.989401	19.947	9.226	24.820
S4532.E1.L1	DS	69.1%	2,577,523,845	78,884,451	99,141,301	110,043,936	46	62	63	54	41.7%	18.7%	34.4%	1,148,250	932,718	959,501	1,130,902	20.690906	17.284	8.494	26.114
S2514.E1.L1	DS	75.8%	2,527,210,551	113,661,363	99,289,207	120,124,073	44	56	56	51	39.6%	21.2%	27.6%	1,149,061	926,540	924,542	1,100,117	26.029809	21.351	8.164	22.906
S1960.E1.L1	DS	93.2%	1,725,743,223	114,318,024	98,726,011	102,690,235	50	62	63	58	43.9%	26.2%	36.0%	1,144,945	987,361	989,363	1,123,767	26.379657	23.066	10.555	25.417
S1496.E1.L1	DS	85.5%	2,516,632,984	110,844,132	116,688,408	104,487,273	44	58	59	54	34.8%	24.7%	33.3%	1,148,075	982,715	1,007,662	1,125,313	33.817423	20.338	11.077	24.524
S2861.E1.L1	DS	94.9%	1,581,288,485	95,125,912	98,601,383	102,898,166	49	56	60	53	21.2%	22.2%	35.6%	1,149,674	963,971	973,089	1,124,139	27.212571	15.530	13.007	28.835
S1507.E1.L1	DS	66.6%	2,190,377,154	112,632,143	92,203,232	122,428,470	46	60	62	55	36.0%	24.2%	34.1%	1,145,533	986,514	962,047	1,127,321	25.511422	24.653	10.813	30.646
S1961.E1.L1	DS	76.2%	2,005,096,673	114,032,076	107,798,886	132,005,549	49	60	63	54	43.0%	25.6%	32.8%	1,144,017	974,391	989,221	1,126,761	25.828512	28.049	12.580	31.813
S2520.E1.L1	DS	87.3%	2,014,245,352	117,091,275	105,749,641	110,176,205	45	58	59	53	40.7%	23.4%	29.5%	1,149,058	936,241	956,714	1,104,061	27.544326	28.105	11.492	24.415
S5319.E1.L1	DS	95.5%	1,630,628,900	112,717,831	96,926,398	99,049,210	43	60	62	53	42.3%	21.9%	34.6%	1,149,058	975,859	972,853	1,125,249	29.167912	28.373	11.294	25.987

Supplemental Table S2: Effectiveness of target enrichment per library after duplicate removal

For each library, we downsampled to 25 million reads which is a typical number generated in a capture experiment, removed duplicates, and computed the average coverage in the specified subset of the genome, divided by the average on the common core of 1,150,639 autosomal SNPs targeted by all three reagents. We use color intensity to indicate the relative efficiencies of coverages of each library (males and females are colored separately for the X Chromosome, and only males are colored for the Y Chromosome). The "Average" column is the number in Table 2.

	Targeted subset of the genome (some categories overlap)	Average (only for males and multipying by 2 for X and Y chromosomes)	# positions (either SNPs or tiled nucleotides)	S2818.Y1.E4.L1	S10872.E1.L4	S10871.E1.L6	S8432.E1.L9	S13982.Y1.E8.L1	S20720.Y1.E1.L1	S20721.Y1.E1.L1	S21299.Y1.E1.L1	S20703.Y1.E1.L1	S1633.E1.L1	S11857.E1.L1	S10871.E1.L1	S1507.E1.L1	S1583.E1.L1	S4532.E1.L1	S5950.E1.L1	S1734.E1.L1	S2514.E1.L1	S1961.E1.L1	S1965.E1.L1	S1496.E1.L1	S2520.E1.L1	S5319.E1.L1	S2949.E1.L7	S4795.E1.L1	S1960.E1.L1	S2861.E1.L1
			**Library type**	SS	SS	SS	SS	SS	DS	DS	DS	DS	DS	DS	DS	DS	DS	DS	DS	DS	DS	DS	DS	DS	DS	DS	DS	DS	DS	DS
			**Molecular sex**	M	M	M	F	F	M	F	F	F	F	M	M	M	M	M	M	M	M	M	M	M	M	M	F	F	F	F
			**Human sequences in shotgun sequencing (%)**	1.2%	4.2%	42%	0.2%	6.9%	0.1%	1.2%	2.0%	6.6%	87%	7.5%	53%	67%	69%	69%	70%	74%	76%	76%	78%	85%	87%	96%	1.7%	79%	93%	95%
**1240k**	SNPs - Affymetrix Human Origins	0.984	597,573	1.02	0.99	0.93	0.99	0.94	1.01	1.05	1.04	1	0.99	1.04	0.984	0.98	0.98	0.97	0.97	0.97	0.97	0.96	0.96	0.96	0.95	0.95	1	0.995	0.99	0.98
**1240k**	SNPs - Illumina 650Y	0.959	660,611	0.95	0.96	0.99	0.98	1.01	0.95	0.94	0.94	0.96	0.96	0.93	0.984	0.95	0.96	0.95	0.96	0.96	0.96	0.96	0.96	0.96	0.96	0.96	0.95	0.945	0.96	0.97
**1240k**	SNPs - Affymetrix 50K	0.392	58,559	0.44	0.41	0.53	0.52	0.45	0.52	0.51	0.43	0.41	0.37	0.4	0.413	0.34	0.36	0.33	0.34	0.34	0.34	0.33	0.33	0.34	0.3	0.3	0.36	0.432	0.37	0.35
**1240k**	SNPs - 1240k phenotypic supplement	1.005	45,969	0.96	0.96	1.27	0.94	0.82	0.7	0.92	1.03	1	0.98	0.99	1.168	1.03	1.01	1.03	1.04	1.03	1.03	1.02	1.02	1.03	1.07	1.06	1	1.104	0.97	0.98
**1240k**	SNPs - 1240k X content	0.978	49,704	0.38	0.36	0.54	0.73	0.87	0.27	0.77	0.7	0.64	0.68	0.35	0.421	0.33	0.34	0.33	0.34	0.34	0.34	0.33	0.34	0.34	0.32	0.32	0.68	0.589	0.69	0.68
**1240k**	SNPs - 1240k Y content	0.974	32,670	0.63	0.69	1.22	0.07	0.04	0.76	0.06	0.08	0.1	0.08	0.6	0.722	0.67	0.68	0.69	0.67	0.69	0.72	0.7	0.73	0.68	0.68	0.7	0.08	0.136	0.09	0.06
**1240k**	SNPs - Twist phenotypic supplement	0.068	94,587	0.06	0.06	0.05	0.07	0.07	0.09	0.08	0.08	0.08	0.06	0.07	0.117	0.06	0.06	0.06	0.06	0.07	0.06	0.06	0.06	0.06	0.06	0.06	0.08	0.067	0.06	0.06
**1240k**	SNPs - Twist Y content	0.446	81,925	0.3	0.32	0.46	0.04	0.02	0.34	0.03	0.04	0.04	0.03	0.29	0.348	0.31	0.32	0.33	0.31	0.32	0.34	0.33	0.34	0.32	0.31	0.32	0.04	0.076	0.04	0.03
**1240k**	SNPs - Arbor ancestral supplement	0.14	852,068	0.13	0.14	0.13	0.14	0.13	0.13	0.15	0.15	0.14	0.14	0.14	0.181	0.13	0.14	0.13	0.13	0.14	0.14	0.13	0.13	0.13	0.13	0.13	0.15	0.188	0.14	0.14
**1240k**	SNPs - Arbor Y supplement	0.15	46,218	0.12	0.12	0.09	0.01	0.01	0.09	0	0	0.01	0	0.12	0.18	0.11	0.11	0.12	0.11	0.11	0.12	0.11	0.11	0.11	0.11	0.11	0	0.039	0	0
**1240k**	Tiling - Mitochondrial DNA	457	16,569	96	27	232	24	155	944	197	83	319	66	30	3748	36	43	62	38	48	43	42	48	45	40	41	75	5757	43	66
**1240k**	Tiling - Twist HAR supplement	0.043	857,339 (3171 HARs)	0.05	0.04	0.05	0.05	0.04	0.01	0.06	0.05	0.05	0.04	0.05	0.108	0.04	0.04	0.03	0.04	0.04	0.04	0.03	0.03	0.03	0.03	0.03	0.05	0.048	0.04	0.04
**1240k**	Tiling - Twist gene sequencing supplement	0.513	2,577 (in three genes)	0.31	0.48	0.48	0.05	0	0	0.34	0.23	0.1	0.67	0.34	0	0.55	0.56	0.82	0.63	0.77	0.81	0.98	0.88	0.59	0.8	0.92	0.83	0	0.76	0.97
**1240k**	Tiling - Twist methylation targets	0.046	80,000 (40,000 CpGs)	0.04	0.04	0.03	0.04	0.02	0.02	0.05	0.05	0.06	0.05	0.05	0.076	0.04	0.05	0.04	0.05	0.05	0.05	0.04	0.04	0.04	0.05	0.05	0.06	0.068	0.04	0.04
**Twist Ancient DNA**	SNPs - Affymetrix Human Origins	1.109	597,573	1.1	1.12	1.03	1.07	1.08	1.11	1.09	1.1	1.1	1.13	1.14	1.082	1.12	1.12	1.12	1.12	1.12	1.12	1.12	1.13	1.12	1.13	1.13	1.13	1.07	1.13	1.13
**Twist Ancient DNA**	SNPs - Illumina 650Y	0.899	660,611	0.91	0.9	0.96	0.94	0.93	0.87	0.93	0.9	0.91	0.89	0.88	0.935	0.89	0.89	0.89	0.88	0.89	0.89	0.88	0.88	0.89	0.87	0.87	0.89	0.935	0.9	0.9
**Twist Ancient DNA**	SNPs - Affymetrix 50K	0.544	58,559	0.59	0.56	0.73	0.69	0.56	0.54	0.63	0.49	0.51	0.5	0.5	0.574	0.53	0.54	0.54	0.5	0.52	0.53	0.5	0.53	0.5	0.44	0.45	0.54	0.638	0.55	0.53
**Twist Ancient DNA**	SNPs - 1240k phenotypic supplement	0.929	45,969	0.89	0.9	0.86	0.87	0.89	0.83	0.89	0.99	0.98	0.94	0.91	0.962	0.93	0.93	0.93	0.96	0.94	0.94	0.95	0.93	0.95	1.01	0.99	0.9	0.988	0.89	0.91
**Twist Ancient DNA**	SNPs - 1240k X content	1.068	49,704	0.42	0.39	0.49	0.85	0.88	0.34	0.83	0.74	0.72	0.73	0.36	0.49	0.38	0.38	0.38	0.36	0.38	0.38	0.37	0.38	0.37	0.35	0.35	0.76	0.677	0.77	0.76
**Twist Ancient DNA**	SNPs - 1240k Y content	0.692	32,670	0.51	0.52	0.52	0.09	0.07	0.43	0.04	0.05	0.09	0.04	0.46	0.528	0.53	0.52	0.53	0.52	0.52	0.53	0.54	0.54	0.52	0.51	0.52	0.03	0.08	0.04	0.03
**Twist Ancient DNA**	SNPs - Twist phenotypic supplement	0.968	94,587	0.84	0.88	0.85	0.86	1.22	1.51	0.88	1.06	1.07	0.99	0.9	0.9	0.93	0.92	0.93	0.98	0.94	0.94	0.97	0.93	0.98	1.09	1.05	0.9	0.825	0.89	0.92
**Twist Ancient DNA**	SNPs - Twist Y content	0.68	81,925	0.52	0.52	0.49	0.05	0.03	0.43	0.02	0.03	0.05	0.02	0.46	0.605	0.53	0.52	0.54	0.51	0.53	0.54	0.54	0.54	0.52	0.51	0.52	0.01	0.052	0.02	0.01
**Twist Ancient DNA**	SNPs - Arbor ancestral supplement	0.157	852,068	0.15	0.15	0.17	0.2	0.2	0.18	0.16	0.16	0.16	0.15	0.14	0.16	0.15	0.15	0.15	0.15	0.15	0.15	0.15	0.15	0.15	0.15	0.15	0.15	0.182	0.15	0.15
**Twist Ancient DNA**	SNPs - Arbor Y supplement	0.624	46,218	0.49	0.49	0.45	0.03	0.01	0.44	0.01	0.01	0.02	0	0.42	0.58	0.5	0.48	0.5	0.47	0.49	0.5	0.51	0.5	0.48	0.47	0.48	0	0.036	0.01	0
**Twist Ancient DNA**	Tiling - Mitochondrial DNA	219	16,569	1	1	4	2	3	108	5	2	32	5	2	2506	3	3	5	2	3	3	3	4	4	3	4	4	3183	3	7
**Twist Ancient DNA**	Tiling - Twist HAR supplement	2.242	857,339 (3171 HARs)	2.34	2.73	2.19	2.13	2.15	1.64	1.43	1.2	1.5	2.32	1.43	2.688	2.52	2.48	2.46	2.37	2.52	2.55	2.5	2.55	2.38	2.26	2.35	2.63	2.234	2.52	2.49
**Twist Ancient DNA**	Tiling - Twist gene sequencing supplement	2.678	2,577 (in three genes)	1.59	2.18	1.95	1	0	2.39	1.4	1.81	3.94	4.55	1.37	0.604	2.76	2.42	2.93	2.7	3.03	3.1	3.34	2.91	2.88	3.68	3.86	4.9	2.322	3.97	4.74
**Twist Ancient DNA**	Tiling - Twist methylation targets	1.599	80,000 (40,000 CpGs)	1.19	1.63	0.99	0.81	1.43	2.24	0.81	1.11	1.71	1.85	0.97	1.508	1.68	1.65	1.68	1.92	1.78	1.8	1.89	1.76	1.92	2.38	2.25	1.7	1.326	1.56	1.62
**Arbor Complete**	SNPs - Affymetrix Human Origins	1.045	597,573	1.01	1.01	0.84	0.98	0.94	1.01	1.05	1.07	1.05	1.09	1.08	0.959	1.06	1.06	1.09	1.08	1.08	1.09	1.09	1.08	1.08	1.1	1.11	1.11	0.946	1.08	1.08
**Arbor Complete**	SNPs - Illumina 650Y	0.963	660,611	0.97	0.97	1.07	1	1.04	0.98	0.96	0.94	0.95	0.94	0.93	1.087	0.94	0.95	0.92	0.93	0.93	0.92	0.93	0.93	0.93	0.91	0.91	0.93	1.153	0.95	0.95
**Arbor Complete**	SNPs - Affymetrix 50K	0.771	58,559	0.85	0.88	1.17	0.89	0.6	0.56	0.77	0.6	0.67	0.71	0.74	1.19	0.79	0.77	0.7	0.7	0.72	0.7	0.71	0.75	0.7	0.61	0.62	0.67	1.216	0.77	0.74
**Arbor Complete**	SNPs - 1240k phenotypic supplement	0.96	45,969	0.92	0.94	1.09	0.94	1.06	0.94	0.9	1.01	1.03	0.9	0.9	1.013	0.93	0.92	0.96	0.93	0.94	0.95	0.95	0.95	0.94	0.97	0.98	0.92	1.156	0.89	0.89
**Arbor Complete**	SNPs - 1240k X content	1.392	49,704	0.57	0.58	0.8	1.01	1.31	0.35	0.92	0.79	0.63	1.01	0.49	0.504	0.54	0.53	0.52	0.51	0.53	0.53	0.53	0.55	0.52	0.49	0.5	1.07	1.005	1.07	1.07
**Arbor Complete**	SNPs - 1240k Y content	1.502	32,670	0.79	0.91	1.41	0.39	0.53	2.54	0.14	0.13	0.46	0.06	0.75	0.759	0.94	0.88	0.96	0.86	0.9	0.99	0.98	1.09	0.86	0.9	0.98	0.04	0.423	0.07	0.04
**Arbor Complete**	SNPs - Twist phenotypic supplement	0.365	94,587	0.13	0.13	0.44	0.42	0.95	1.09	0.35	0.51	1.1	0.16	0.25	1.203	0.15	0.15	0.16	0.15	0.15	0.13	0.13	0.14	0.15	0.16	0.15	0.14	1.078	0.15	0.15
**Arbor Complete**	SNPs - Twist Y content	1.182	81,925	0.62	0.72	0.97	0.24	0.37	2.32	0.08	0.07	0.22	0.04	0.59	0.639	0.74	0.69	0.78	0.68	0.73	0.81	0.8	0.89	0.68	0.72	0.8	0.03	0.298	0.04	0.02
**Arbor Complete**	SNPs - Arbor ancestral supplement	0.695	852,068	0.64	0.6	0.89	0.89	0.81	0.8	0.78	0.87	0.94	0.62	0.67	1.042	0.6	0.6	0.6	0.6	0.59	0.58	0.58	0.58	0.6	0.61	0.59	0.59	0.936	0.6	0.6
**Arbor Complete**	SNPs - Arbor Y supplement	1.06	46,218	0.57	0.68	0.85	0.12	0.24	2.24	0.04	0.03	0.06	0.02	0.53	0.566	0.7	0.63	0.74	0.63	0.7	0.78	0.76	0.85	0.63	0.66	0.76	0.01	0.184	0.02	0.01
**Arbor Complete**	Tiling - Mitochondrial DNA	3250	16,569	159	123	395	42	292	4697	144	61	873	359	45	23387	264	217	605	200	369	408	396	528	347	382	450	413	51757	309	517
**Arbor Complete**	Tiling - Twist HAR supplement	0.265	857,339 (3171 HARs)	0.1	0.1	0.38	0.41	0.24	0.46	0.23	0.26	0.93	0.11	0.17	1.445	0.1	0.11	0.1	0.1	0.1	0.08	0.08	0.08	0.1	0.09	0.08	0.08	1.032	0.11	0.1
**Arbor Complete**	Tiling - Twist gene sequencing supplement	0.293	2,577 (in three genes)	0.07	0.04	0	0	0	0	0.19	0	4.08	0.23	0.32	1.818	0.07	0.08	0.09	0.08	0.08	0.05	0.04	0.04	0.04	0.09	0.09	0.14	0	0.12	0.15
**Arbor Complete**	Tiling - Twist methylation targets	0.197	80,000 (40,000 CpGs)	0.04	0.04	0.19	0.13	0.26	0.8	0.19	0.26	0.91	0.08	0.11	0.689	0.06	0.06	0.07	0.07	0.06	0.05	0.05	0.05	0.07	0.08	0.07	0.06	0.744	0.07	0.06

Supplemental Table S3: Effectiveness of target enrichment before duplicate removal

For each library, we downsampled to 25 million reads which is a typical number generated in a capture experiment, and computed the average coverage in the specified subset of the genome, divided by the average on the common core of 1,150,639 autosomal SNPs targeted by all three reagents. The lines for autosomal regions show the mean of these ratios across all 27 libraries. The lines for X and Y Chromosome regions show the average across males, after multiplying by a factor of two to show effectiveness of enrichment on a per-genome-copy basis (males are haploid on the sex chromosomes versus diploid on the autosomes, so the factor of two adjusts for copy number difference). This table show results before duplicate removal, and Table 2 in the main text shows results after duplicate removal.

Targeted subset of the genome (some categories overlap)	# positions (either SNPs or tiled nucleotides)	1240k coverage (vs. core set)	Twist coverage (vs. core set)	Arbor coverage (vs. core set)
**SNPs**				
Affymetrix Human Origins	597,573	0.955	1.121	1.062
Illumina 650Y	660,611	0.969	0.89	0.948
Affymetrix 50K	58,559	0.351	0.521	0.751
1240k phenotypic supplement	45,969	1.01	0.928	0.936
1240k X content	49,704	0.946	1.034	1.372
1240k Y content	32,670	1.108	0.720	1.356
Twist phenotypic supplement	94,587	0.054	0.971	0.226
Twist Y content	81,925	0.488	0.704	1.072
Arbor ancestral supplement	852,068	0.13	0.149	0.654
Arbor Y supplement	46,218	0.140	0.646	0.986
**Tiling nucleotides**				
Mitochondrial DNA	16,569	171	46	2450
Twist HAR supplement	857,339 (3171 HARs)	0.03	2.941	0.162
Twist gene sequencing supplement	2,577 (in three genes)	0.692	4.035	0.175
Twist methylation targets	80,000 (40,000 CpGs)	0.038	2.108	0.111

Supplemental Table S4: Comparison of experimental settings across assays

Hybridization	1240k	Arbor	optimized Twist settings for Ancient DNA	original Twist settings from 'Protocol_NGS_HybridizationTE_31OCT19_Rev1'
number of libraries	1	1	1	≤ 8
library DNA amount in μg	1	1	1	≤ 1.5
probe length in bp	52	-	80	120
probe volume in μl	4	4	1 (+0.167*)	4 (+4)
hybridization buffer volume in μl	20	20	5	20
total hybridization volume in μl	34	34	18 (18.167)	28
hybridization temperature in °C	73	70	62	70
hybridization time in thermocycler in hours	≥ 16	≥ 16	≥ 16	≥ 16
**Capture and washes**				
Streptavidin bead amount in μl	30	30	300	100
number of wash buffers	3	3	2	2
number of washes	5	5	4 (7**)	4
number of stringent washes	3	3	3 (6**)	3
temperature in °C of stringent washes	57	55	49	48
number of PCR cycles	30	20	23	5-16

* 0.167 μl MT probes (Twist mitochondrial panel, high conc, 104562)

** number of washes is increased for automated proceasing due to the smaller volumes used in plates

Supplemental Figure S1: Coverage on autosomal SNP targets as a function of sequencing depth

For 10 libraries (5 double stranded – DS, and 5 single stranded libraries - SS) with varying varying percentages of human sequences before enrichment (0.1-86.7%) we show the average coverage at the shared set of 1.15 million autosomal SNPs targets at different levels of sequencing depth (based on downsampling). Results are for the 10 libraries spanning a wide range of library characteristics, after removal of duplicated sequences.

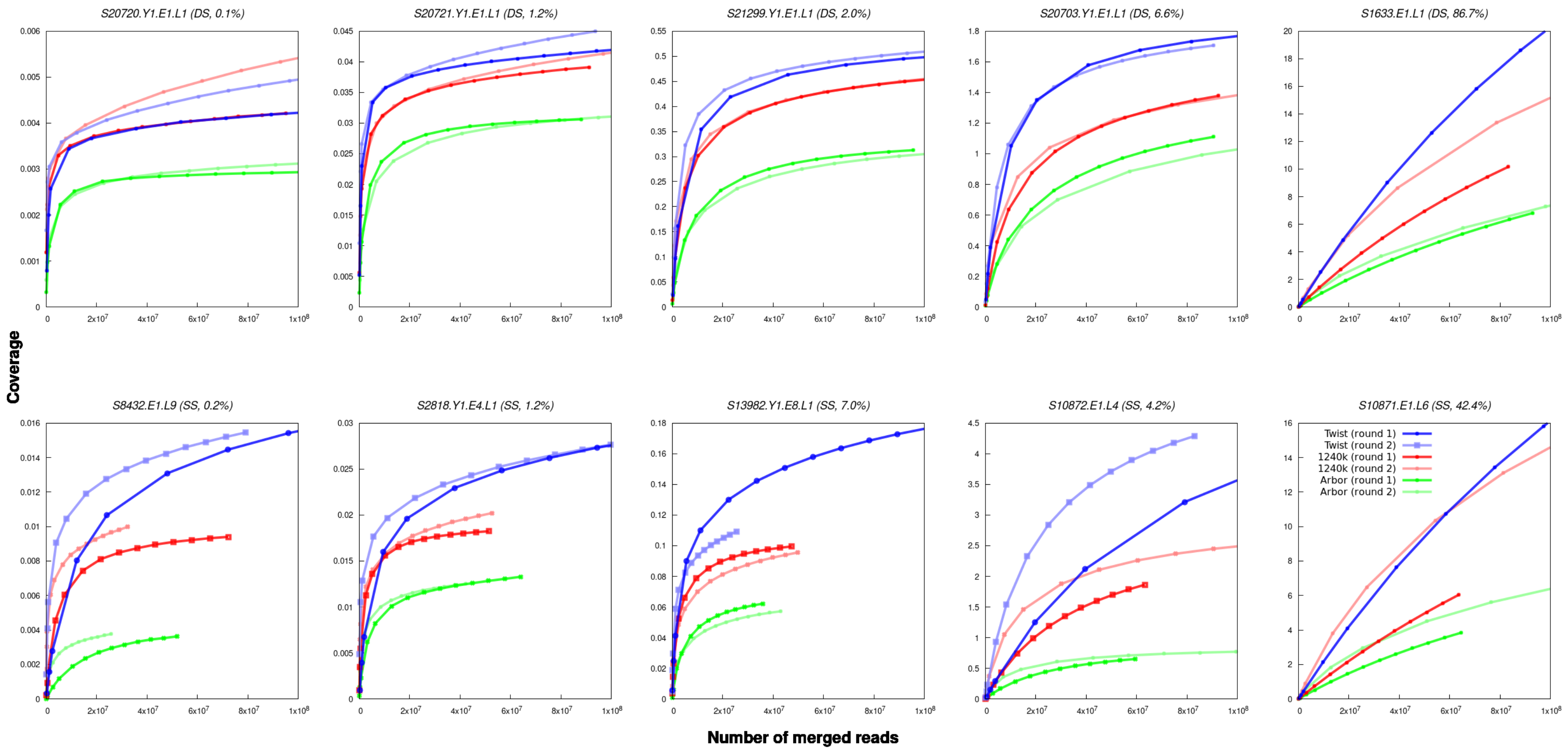

Supplemental Figure S2: Distribution of %GC and sequence lengths in 10 library experiment

For 10 libraries (5 double stranded – DS, and 5 single stranded libraries - SS) with varying percentages of human sequences before enrichment (0.1-86.7%) we show (A) the distribution of %GC, and (B) sequence lengths, for data downsampled to 25 million merged sequences. Results are after duplicate removal; results without duplicate removal are similar and not shown.

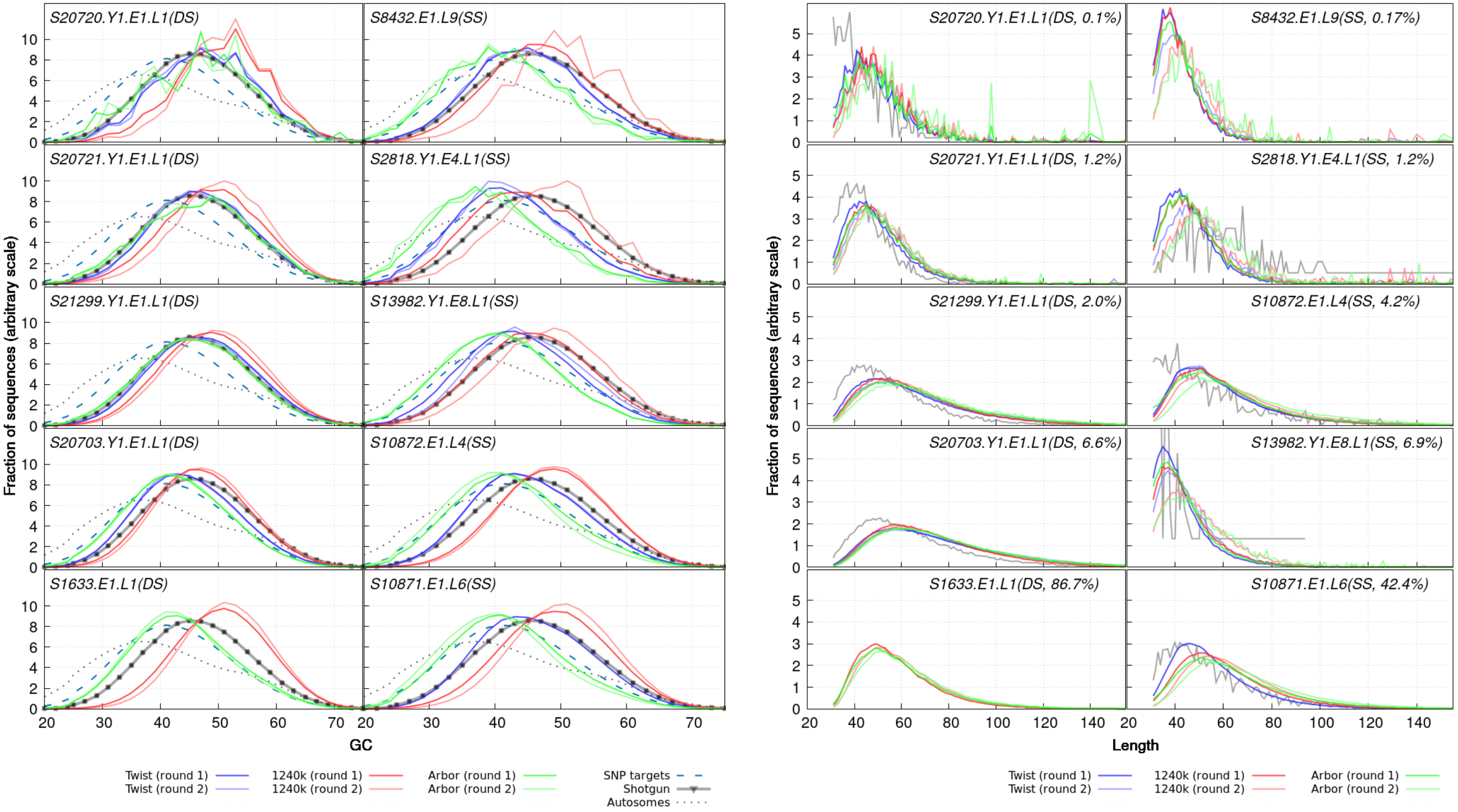

A **B**

Supplemental Figure S3: Distribution of sequence lengths in 17 library experiment

For 17 double-stranded (DS) libraries with the specified percentages of human sequences before enrichment, we show the distribution of sequence lengths for data downsampled to 25 million merged sequences. Results are after duplicate removal; results without duplicate removal are similar and not shown.

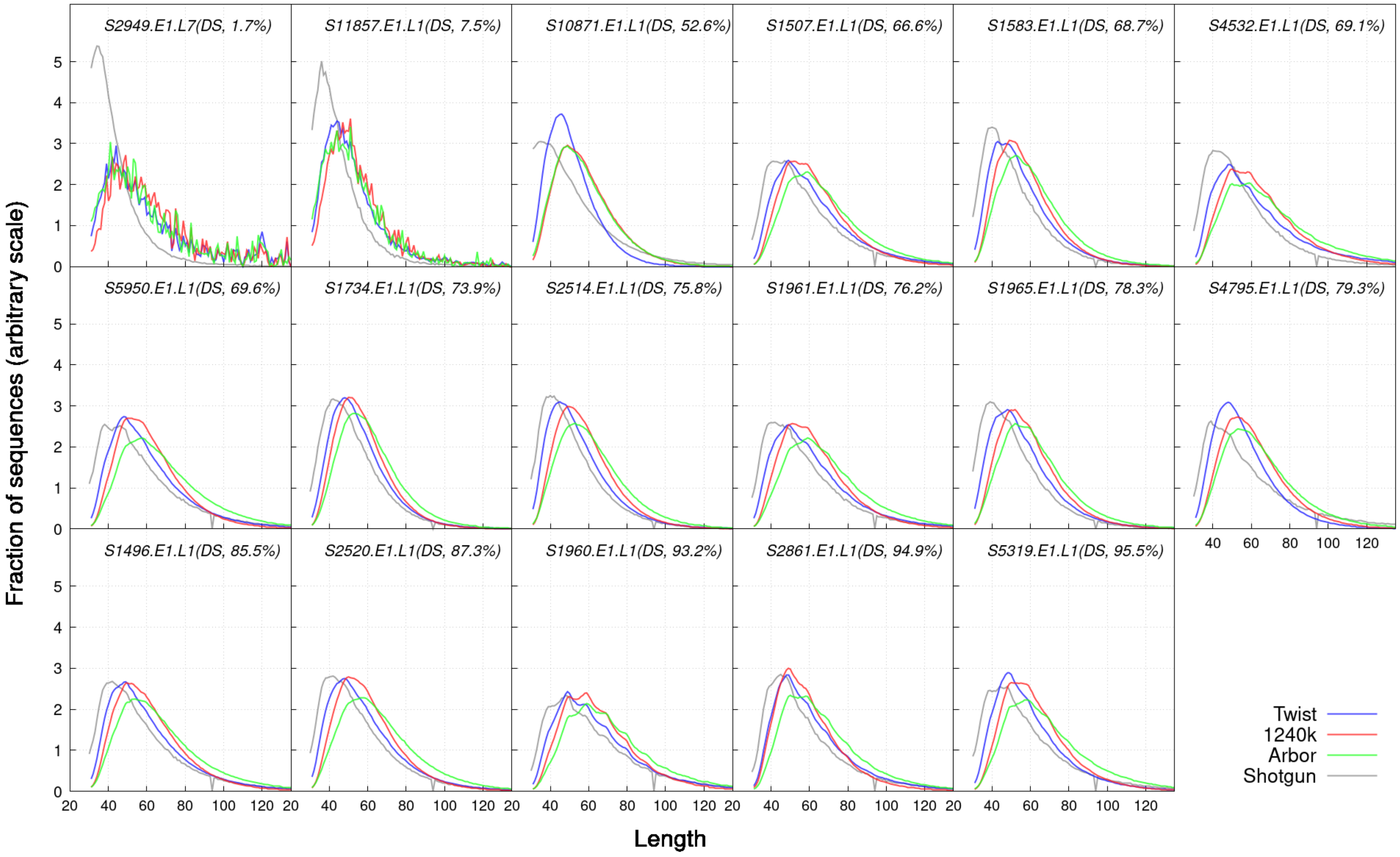

Supplemental Figure S4: Distribution of %GC in 17 library experiment

For 17 double-stranded (DS) libraries with the specified percentages of human sequences before enrichment, we show the distribution of %GC for data downsampled to 25 million merged sequences. Results are after duplicate removal; results without duplicate removal are similar and not shown.

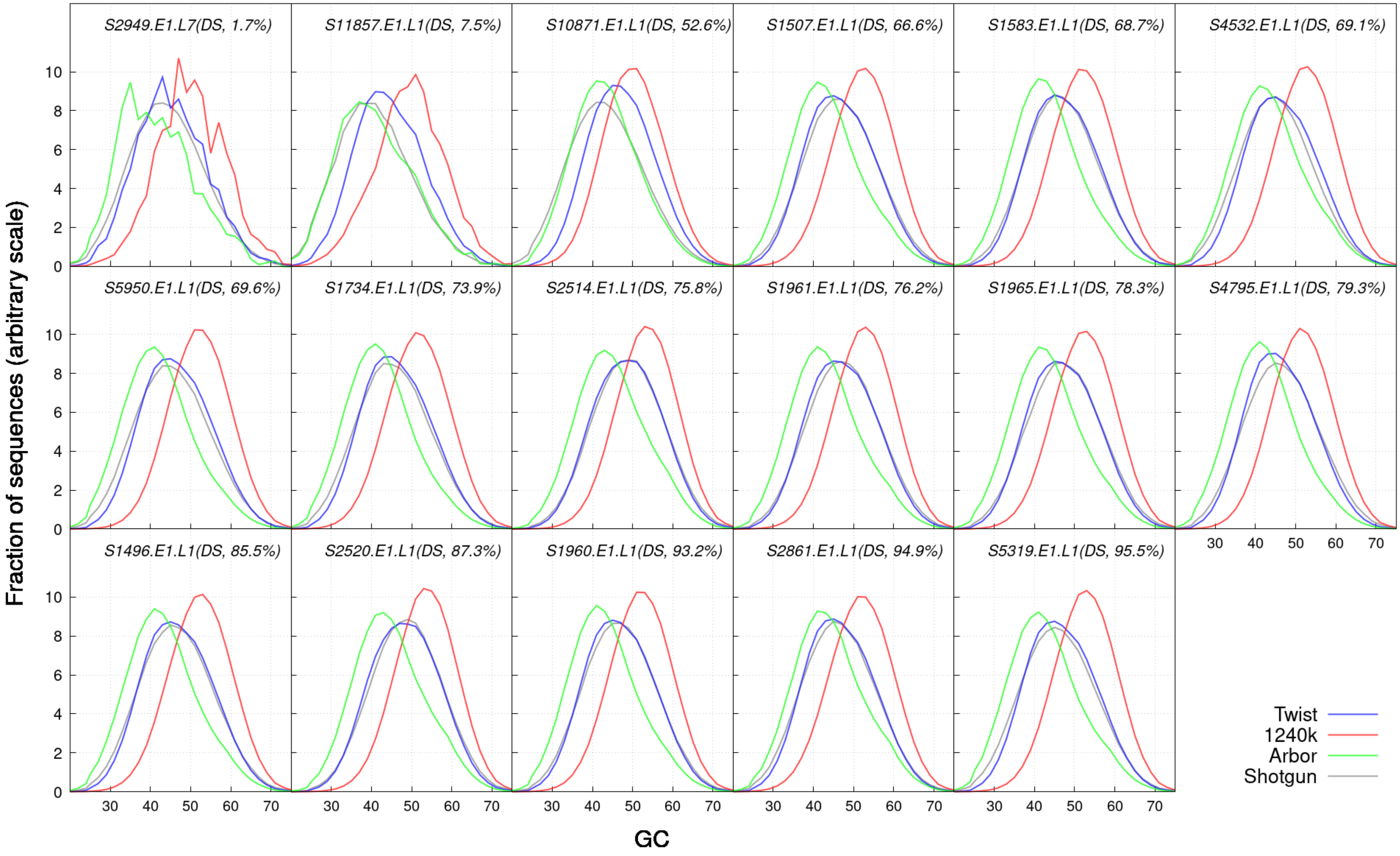

Supplement: Supplemental Material [file supp_gr.276728.122_Supplemental_Material.pdf]
